# Supplementary material for: Two-epoch cross-sectional case record review protocol comparing quality of care of hospital emergency admissions at weekends versus weekdays
Source: BMJ Open. 2017 Dec 22;7(12):e018747. doi: 10.1136/bmjopen-2017-018747 (PMC5770964; doi:10.1136/bmjopen-2017-018747)
Supplement: Supplementary file 1 [file bmjopen-2017-018747supp001.pdf]

APPENDICES

**Appendix 1: HiSLAC Case Record Review Data Collection**

**On registration of each reviewer, we will allocate an unique Reviewer code which will function as log-in user name and link to the details below:**

| | |
|---|---|
| Name: | |
| Email: | |
| Phone number: | |
| Date of Birth (age) | |
| Gender | |
| Grade: Specialist Registrar training years 5-7, or Consultant | |
| Year of graduation: | |
| Current speciality/specialities (CCT programmes) –drop-down list | |

**TO BE RECORDED AUTOMATICALLY AT THE BEGINNING OF EACH CASE RECORD REVIEW**

- Patient unique study number:
- Reviewer's Code
- Date and time of the review:

**DATA COLLECTION**

| 1. **HES DATA EXTRACTED BY PROJECT TEAM BEFORE CASE RECORD REVIEW (not made available to reviewers)** | |
|---|---|
| Sex M/F | |
| Age at hospital admission | |
| Date and Time of patient's first arrival at hospital (ED or other primary receiving ward). | |
| Duration of stay in ED (interval between arrival at hospital and time of admission, hrs & mins) | |
| Time, date and day of admission | |
| Length of hospital stay (days) | |
| Primary admitting diagnosis | |
| Comorbid disease (Charlson) | |
| Hospital Outcome: death or survival | |

| **2. CLINICAL DATA EXTRACTED BY CASE RECORD REVIEWER (independently for duplicate reviews)** | |
|---|---|
| 1. **PRE-ADMISSION PHASE INCLUDING EMERGENCY DEPARTMENT** | |
| Source of admission:<br>• Own home<br>• Nursing or residential care home<br>• Another hospital<br>• No fixed abode<br>• No information available | |
| Patient condition immediately before the illness that led to this admission. | |

| | |
|---|---|
| • Independent<br>• Needing help with some activities of daily living (ADLs)<br>• Dependant on others for most/all ADLs including personal hygiene<br>• Unable to determine; no relevant information in notes | |
| Referral mechanism:<br>• Self-presentation to ED (walk-in/own transport)<br>• 999/ambulance transfer to ED<br>• GP or deputising service referral<br>• Unable to determine | |
| Admission pathway:<br>Was the patient initially assessed in ED or any other short term emergency pre-admission assessment unit (e.g. Clinical Decision Unit, Ambulatory care, Medical or Surgical Assessment Unit, etc.), or was the patient admitted directly to an acute ward (AMU, general or specialty ward)?<br>• ED/pre-admission area<br>• Direct admission to acute ward<br>• Unable to determine | |
| 2. **POST-ADMISSION PHASE** | |
| Location immediately following admission:<br>• Clinical Decision Unit or short stay ward<br>• Acute Medical Unit (AMU/MAU)<br>• General medical ward<br>• Medical sub-specialities including high care (eg: Coronary Care Unit, Renal Unit, Respiratory, Haematology, Oncology)<br>• Older People's Medicine/Elderly Care Unit<br>• Rehabilitation<br>• Critical Care Unit / Intensive Care Unit (including High Dependency)<br>• General Surgery (including surgical assessment/operating theatre)<br>• Other (please specify):<br>• Unable to determine ward type | |
| Was this an appropriate type of ward for the patient's condition?<br>• Yes, definitely appropriate<br>• Probably appropriate<br>• No<br>• Unable to determine | |
| Were vital signs recorded for calculation of a National Early Warning Score in the first 12 hours following admission?<br>• Yes, full vital signs and a NEWS recorded<br>• Full vital signs, but NEWS not documented<br>• Some vital signs not documented, no NEWS<br>• No evidence of vital signs or NEWS | |
| Initial Consultant Review in the first 24 hours following admission:<br>• Consultant review documented [REVIEWER TO RECORD TIME AND DATE]<br>• Probable consultant review but status of doctor uncertain [REVIEWER TO RECORD TIME AND DATE]<br>• Consultant review, time not documented, but case record suggests < 14 hrs after admission<br>• Consultant review, time not documented, but case record suggests > 14 hrs after admission | |

| | |
|---|---|
| • Unlikely that consultant review occurred during first 24 hours<br>• No evidence for consultant review in first 24 hours | |
| Palliative and end-of-life care (within first 7 days): were discussions held or decisions made to limit treatment, forego resuscitation (DNACPR), or refer to palliative care?<br>• No: not required, patient appropriately for full treatment<br>• No, but would probably have been appropriate to consider some form of treatment limitation<br>• No, but would definitely have been appropriate to limit treatment.<br>• Yes: time and date of discussion or decision to limit treatment<br>  ○ Yes, appropriate decision<br>  ○ Yes, but patient might have benefited from escalation<br>  ○ Yes, but likely inappropriate decision, patient should have been considered for full escalation<br>• If referral to palliative care, time and date referral made: | |
| Admission avoidance: Could admission to hospital have been avoided given optimal care in the community (eg: primary care, Hospital at Home, palliative care etc)?<br>• Yes<br>• Possibly<br>• No | |
| Free Text Comments: Anything else you want to mention about this case? | |
| **3. ASSESSMENT OF CARE QUALITY** | |
| Errors in care:<br>Does the case record contain evidence of one or more errors in care defined as '*the failure of a planned action to be completed as intended or use of a wrong, inappropriate, or incorrect plan to achieve an aim*' (This may include events identified above). If Yes, please complete next table, errors in healthcare:<br>• Description of event<br>• Where did the event occur?<br>• When did the event occur?<br>• Category of event<br>• Associated with adverse event?<br>• If yes, grade preventability | |
| ***Proceed to next table, errors in care*** | |

**Errors in care. Please complete the following table using the categories listed below this table**

- **An error in care is defined as '**the failure of a planned action to be completed as intended or use of a wrong, inappropriate, or incorrect plan to achieve an aim**'.** Errors may or may not have direct consequences for the patient, but they should have the potential to have adverse consequences. To identify errors**,** consider what an acceptable standard of healthcare would be for this patient, and describe whether (and how) the healthcare they received fell below this acceptable standard (whether through commission, omission, delay or incorrect actions). **Only one error should be entered per row.**
- **Where did the error occur (Appendix 1.1)?**
- **When did the error occur (enter date and time)**
- **What was the category of error (Appendix 1.2)? More than one may be chosen, in which case please list the most important first.**
- **Was the error associated with an adverse event (AE)? We define an AE as '***An event that results in unintended harm to the patient by an act of commission or omission rather than by the underlying disease or condition of the patient'*. **Responses: Yes / No / Insufficient evidence**
- **Preventability: If in your opinion the error resulted in an adverse event, to what extent was the adverse event preventable? Please use the grading below.**
    1. Virtually no evidence for preventability.
    2. Slight to modest evidence of preventability.
    3. Possibly preventable, but not very likely (less than 50–50, but close call).
    4. Probably preventable (more than 50–50, but close call).
    5. Strong evidence for preventability
    6. Virtually certain evidence of preventability.

| **Describe each error in your own words. Please describe what should have happened AND what did happen.** | **Where did the error occur? (*Appendix 1.1*)** | **When did the error occur? (*Date, time*)** | **Category of error (*Appendix 1.2*)** | **Was the error associated with an adverse event? (*Y / N / insufficient evidence*)** | **To what extent was the adverse event preventable (*Appendix 1.3, grade 1-6*)?** |
|---|---|---|---|---|---|
| *Example: "First dose of IV antibiotics should have been given immediately but was not given until three hours after prescribed"* | *B* | *Date and time* | *B* | *N* | *n/a* |
| *Example: Inappropriate administration of thrombolysis for an assumed minor pulmonary embolism, when in fact the patient had sustained a pulmonary haemorrhage.* | *C* | *Date & time* | *A & B* | *Y* | *4* |
| *Example: no vital signs / NEWS records were documented in the first 12 hours following transfer from the AMU to the ward. The patient suffered a cardiac arrest from which she could not be resuscitated.* | *D* | *Date & time* | *F* | *Y* | *5* |
| *Example: a single dose of a NSAID was given to this patient with AKI; the prescription was cancelled by the consultant the following morning.* | *D* | *Date & time* | *B* | *N* | *n/a* |
| | | | | | |

| **Describe each error in your own words. Please describe what should have happened AND what did happen.** | **Where did the error occur? (*Appendix 1.1*)** | **When did the error occur? (*Date, time*)** | **Category of error (*Appendix 1.2*)** | **Was the error associated with an adverse event? (*Y / N / insufficient evidence*)** | **To what extent was the adverse event preventable (*Appendix 1.3, grade 1-6*)?** |
|---|---|---|---|---|---|
| | | | | | |
| | | | | | |
| | | | | | |

| Global assessment of quality of care |
|---|
| To what extent did this patient receive best practice care? (select one only) |
| • Completely |
| • Substantially |
| • Partially |
| • Very little |
| • Not at all |

| How confident are you in your judgement of global quality? (select one only) |
|---|
| ☐ Completely confident |
| ☐ Substantially confident |
| ☐ Partially confident |
| ☐ Slightly confident |
| ☐ Not at all confident |

| **To what extent was the adverse event preventable?** |
|---|
| 1. Virtually no evidence for preventability. |
| 2. Slight to modest evidence of preventability |
| 3. Possibly preventable, but not very likely (less than 50–50, but close call) |
| 4. Probably preventable (more than 50–50, but close call) |
| 5. Strong evidence for preventability |
| 6. Virtually certain evidence of preventability |
| n/a = not applicable, no adverse event |

**Appendix 2: Reference Materials**

| **1.1 Location Of Error: Where did the error occur?** |
|---|
| A. Outside hospital (primary care, ambulance, etc.) |
| B. In the ED or linked area before admission |
| C. AMU or equivalent area |
| D. Acute ward (other than AMU) |
| E. Speciality ward (ICU/HDU, coronary care, renal, respiratory, elderly care, rehab) |
| F. Diagnostic area, radiology |

| **1.2 Error Typology** | ***Examples only (not exhaustive – not for recording)*** |
|---|---|
| Assessment, investigation or diagnosis | Physical examination and history taking incomplete<br>Pressure ulcer risk not assessed/incorrectly assessed<br>VTE risk assessment not completed/incorrectly completed<br>Falls history/vulnerability to falls not identified<br>Swallowing safety not assessed/incorrectly assessed<br>Tests and investigations missed/delayed/wrong<br>Diagnosis missed/delayed/wrong<br>Failure to assess comorbidities or frailty |

| | |
|---|---|
| Medication | Over- or under-hydration<br>Oxygen supply wrong/delayed/omitted<br>Allergic/anaphylactic reaction to any medication<br>Anticoagulants/antiplatelets wrong/delayed/omitted<br>Antibiotics wrong/delayed/omitted<br>Insulin or other diabetes medication wrong/delayed/omitted<br>Opiates wrong/delayed/omitted<br>Sedatives/hypnotics/antipsychotics wrong/delayed/omitted<br>Steroids wrong/delayed/omitted<br>NSAID wrong/delayed/omitted<br>Diuretics wrong/delayed/omitted<br>Antihypertensives wrong/delayed/omitted<br>Cardiovascular medications wrong/delayed/omitted<br>Chemotherapy wrong/delayed/omitted |
| Treatment and management plan | Appropriate medical/surgical treatment not planned<br>Avoidable delay/omission of planned medical/surgical treatment<br>Inappropriate/unnecessary medical/surgical treatment given<br>Inappropriate ceiling of care<br>Omitted/delayed/wrong treatment from AHPs<br>Acquired pressure ulcer: prevention below acceptable standard<br>Acquired pressure ulcer despite apparently acceptable standard of prevention<br>Slip/trip/fall: prevention plan below acceptable standard<br>Slip/trip/fall despite apparently acceptable standard of falls prevention |

| | |
|---|---|
| | Developed VTE: prophylaxis below acceptable standard<br>Developed VTE despite apparently acceptable standard of VTE prophylaxis |
| Infection control | Surgical wound infection<br>Infection from invasive procedure other than surgery<br>Other healthcare associated wound infection *(e.g. infected ulcer)*<br>Infection from indwelling device *(catheter, central lines, etc.)*<br>Healthcare associated clostridium difficile<br>Healthcare- /device-associated MRSA bloodstream infection<br>Other bloodstream infection (not MRSA)<br>Healthcare associated pneumonia/chest infection (including aspiration)<br>Healthcare associated norovirus/D&V |
| Invasive procedures | Avoidable delay in undertaking procedure<br>Inadequate pre-procedure assessment/preparation<br>Complication of anaesthesia/sedation including airway management<br>Complication of operative procedure *(e.g. perforation, haemorrhage)*<br>Complication of invasive procedure *(e.g. perforation, haemorrhage)* |

| | |
|---|---|
| Monitoring | Vital signs monitoring.<br>Fluid intake/output.<br>Nutritional intake.<br>Delay in initiating resuscitation |
| Cardiopulmonary resuscitation | Delay in initiating cardiopulmonary resuscitation<br>Inappropriate resuscitation<br>Airway management<br>Resuscitation equipment |
| Communication, documentation | Resuscitation status not documented<br>Relatives not informed of resuscitation status<br>Referral to specialist medical team not implemented<br>Incorrect information in correspondence, referral or handover |
| I. Other problem | Describe in free text |

| **1.3: Grading Of Preventability Of Adverse Event:** |
|---|
| 1. Virtually no evidence for preventability. |
| 2. Slight to modest evidence of preventability. |
| 3. Possibly preventable, but not very likely (less than 50–50, but close call). |
| 4. Probably preventable (more than 50–50, but close call). |
| 5. Strong evidence for preventability |
| 6. Virtually certain evidence of preventability. |

| **1.4: Global assessment of quality of care** |
|---|
| To what extent did this patient receive best practice care? (select one only) |

| |
|---|
| • Completely |
| • Substantially |
| • Partially |
| • Very little |
| • Not at all |
