# Supplementary material for: Two-epoch cross-sectional case record review protocol comparing quality of care of hospital emergency admissions at weekends versus weekdays
Source: BMJ Open. 2017 Dec 22;7(12):e018747. doi: 10.1136/bmjopen-2017-018747 (PMC5770964; doi:10.1136/bmjopen-2017-018747)
Supplement: Author's manuscript [file bmjopen-2017-018747.draft_revisions.pdf]

BMJ Open is committed to open peer review. As part of this commitment we make the peer review history of every article we publish publicly available.

When an article is published we post the peer reviewers' comments and the authors' responses online. We also post the versions of the paper that were used during peer review. These are the versions that the peer review comments apply to.

The versions of the paper that follow are the versions that were submitted during the peer review process. They are not the versions of record or the final published versions. They should not be cited or distributed as the published version of this manuscript.

BMJ Open is an open access journal and the full, final, typeset and author-corrected version of record of the manuscript is available on our site with no access controls, subscription charges or pay-per-view fees (http://bmjopen.bmj.com).

If you have any questions on BMJ Open's open peer review process please email editorial.bmjopen@bmj.com

BMJ Open

A Two-Epoch Cross-Sectional Case Record Review Protocol Comparing Quality Of Care Of Hospital Emergency Admissions At Weekends Versus Weekdays

Journal:	*BMJ Open*
Manuscript ID	bmjopen-2017-018747
Article Type:	Protocol
Date Submitted by the Author:	20-Jul-2017

Complete List of Authors:	Bion, Julian; University of Birmingham, Intensive Care Medicine<br>Aldridge, Cassie; University of Birmingham<br>Girling, Alan; University of Birmingham, Institute of Applied Health Research<br>Rudge, Gavin; University of Birmingham, School of Health and Population Sciences<br>Beet, Chris; University Hospitals Birmingham NHS Foundation Trust<br>Evans, Tim; NHS Improvement<br>Temple, R.; Heart of England NHS Foundation Trust, Renal Medicine<br>Roseveare, Chris; Southern Health NHS Foundation Trust<br>Clancy, Mike; University Hospital Southampton NHS Foundation Trust<br>Boyal, Amunpreet; University Hospitals Birmingham NHS Foundation Trust<br>Tarrant, Carolyn; University of Leicester<br>Sutton, Elizabeth; University of Leicester<br>Sun, Jianxia; University Hospitals Birmingham NHS Foundation Trust<br>Rees, Peter; Member of the Academy of Medical Royal Colleges Patient Liaison Group<br>Mannion, Russell; University of York, Management School<br>Chen, Yen-Fu; University of Warwick, Warwick Medical School<br>Watson, Samuel; University of Warwick, Warwick Medical School;<br>Lilford, Richard; University of Warwick, Division of Health and Population Sciences

**Primary Subject Heading**:	Health services research
Secondary Subject Heading:	Health policy
Keywords:	Hospital, weekend, quality, case record review, consultants

SCHOLARONE™ Manuscripts

A Two-Epoch Cross-Sectional Case Record Review Protocol Comparing Quality Of Care Of Hospital Emergency Admissions At Weekends Versus Weekdays

Authors: The HiSLAC collaboration

Writing Committee: Bion JF*, Aldridge C, Girling A, Rudge G, Beet C, Evans T, Temple M, Roseveare P, Clancy M, Boyal A, Tarrant C, Sutton E, Sun J, Rees P, Mannion R, Chen Y-F, Watson S, Lilford R, on behalf of the HiSLAC collaboration.

1. Primary author

*Corresponding author details:
Professor Julian Bion FRCP FRCA FFICM MD
University Department of Anaesthesia & Critical Care Office 1,
Ground Floor East Heritage Building (Queen Elizabeth Hospital)
Mindelsohn Way, Edgbaston,
Birmingham B15 2TH
Tel: 0121 371 6816
Email: J.f.bion@bham.ac.uk

Acknowledgement of Funding: This project was funded by the National Institute for Health Research, Health Services and Delivery Research Programme (project number 12/128/17). The views and opinions expressed therein are those of the authors and do not necessarily reflect those of the HS&DR, NIHR, NHS or the Department of Health.

ABSTRACT

Introduction: The mortality associated with weekend admission to hospital (the 'weekend effect') has for many years been attributed to deficiencies in quality of hospital care, often assumed to be due to suboptimal senior medical staffing at weekends. This protocol describes a case note review to determine whether there are differences in care quality for emergency admissions to hospital at weekends compared with weekdays, and whether the difference has reduced over time as health policies have changed to promote seven-day services.

Methods and analysis: Cross-sectional two-epoch case record review of 20 acute hospital Trusts in England. Anonymised case records of 4000 emergency admissions to hospital, 2000 at weekends and 2000 on weekdays, covering two epochs (financial years 2012-13, and 2016-17). Admissions will be randomly selected across the whole of each epoch from Trust electronic patient records. Following training, structured implicit case reviews will be conducted by senior registrars (senior residents) in acute medical specialities (60 case records per reviewer), and limited to the first seven days following hospital admission. The co-primary outcome measures are error rates and a global assessment of care quality on a Likert scale. Error rates will be analysed using mixed effects logistic regression models, and care-quality using ordinal regression methods. Secondary outcomes include error typology;

For peer review only - http://bmjopen.bmj.com/site/about/guidelines.xhtml

error-related adverse events; and any correlation between error rates and staffing. The data will also be used to inform a parallel health economics analysis.

Ethics and dissemination: The project has received ethics approval from the South West Wales Research Ethics Committee: Reference 13/WA/0372. Informed consent is not required for accessing anonymised patient case records from which patient identifiers had been removed. The findings will be disseminated through peer-reviewed publications in high-quality journals and through local HiSLAC leads at the 121 hospitals that make up the HiSLAC Collaborative.

Methodological Strengths and Limitations

- Difference-in-difference analysis minimises bias from variations in case- mix or institutional characteristics, and allows differentiation of secular trends from the effects of the intervention (specialist intensity).
- Structured implicit case record review encourages a standardised approach to assessment, while permitting reviewers to exercise expert clinical judgement.
- Inter-assessor variation will be measured through duplicate review of 800 case records, and minimised though standardised training.

For peer review only

For peer review only - http://bmjopen.bmj.com/site/about/guidelines.xhtml

INTRODUCTION:

The mortality associated with weekend admission to hospital (the 'weekend effect') has for many years been attributed to deficiencies in quality of hospital care, usually linked to perceptions that senior medical staffing at weekends was suboptimal [1]. The weekend effect has been used as a justification for introducing seven-day services in England [2]. Seven-day services require hospital Trusts in England to meet particular standards for increasing the intensity of weekend senior medical staffing [3]. However, there is little evidence that deficiencies in medical staffing cause the weekend effect. Preliminary research by the High-intensity Specialist Led Acute Care (HiSLAC) collaboration [www.hislac.org] did not identify an association between weekend-weekday specialist intensity differences and weekend-weekday admission mortality rate differences across the English NHS [4]. However, this was a cross-sectional survey in which no hospital Trust achieved parity between weekend and weekday specialist staffing intensity.

Other potential causes of the weekend effect might include patient factors (severity of illness and case mix including imperfect case mix adjustment), contextual factors (resource allocation, health policy, secular trends), and care processes (quality of care). Several studies have identified greater severity of illness amongst weekend admissions [5,6,7,8,9], while another reports increased mortality associated with weekend discharge [10]: these suggest a potential community contribution to the weekend effect. Evidence that quality of care in hospital might be worse at weekends comes from an analysis of an Australian regional voluntary critical incident reporting system [11], but this did not take into account the potential for severity of illness to enhance the opportunity for error, and did not characterize the nature of the critical incidents.

If suboptimal consultant ('specialist') staffing were indeed a cause for the weekend effect, as implied by the 7-day standards policy initiative, there are several ways this might be revealed. First, health care error rates might be higher amongst patients admitted at weekends; second, error typology would include those most likely to be mitigated by the presence of senior physicians (diagnostic accuracy, treatment specificity and timeliness of care); third, a positive association might be expected between the difference in weekend:weekday error rates and the difference in weekend:weekday specialist intensities, indicating that at weekends less reliable care is associated with reduced senior physician presence; and fourth, that weekend performance and staffing indices improve with the introduction of 7-day services focused on maximizing specialist staffing.

We describe here a protocol for comparing quality of care given to patients admitted as emergencies to 20 hospital Trusts at weekends and on weekdays during two epochs, representing periods before (2012-2013) and during (2016-17) the implementation of NHS England's seven-day services standards. The outputs from this protocol will be integrated with parallel HiSLAC research workstreams in specialist intensity [4], a systematic review and framework synthesis [12], ethnography [13], and health economics [14].

For peer review only - http://bmjopen.bmj.com/site/about/guidelines.xhtml

Aims and objectives

Using retrospective review of case records from 20 hospital Trusts during two time epochs, this study aims to determine whether there is a difference in quality of care offered to patients undergoing emergency admission to hospital at weekends compared with weekdays.

The main objectives are:

1. to compare rates of errors and differences in care quality between weekend and weekday admissions
2. to examine prevalent error types for weekend and weekday admissions
3. if a difference in error rates or care-quality between weekend and weekday admissions is found, to examine whether the difference has changed between the two epochs
4. to inform the Bayesian model proposed in our parallel health economics model [14]

A secondary objective is to correlate excess error rates for weekend admissions with deficiencies in specialist staffing within each Trust as measured by weekend:weekday differences in specialist hours per 10 emergency admissions (EAs).

METHODS AND ANALYSIS

We will conduct a cross-sectional two-epoch comparison of care quality received by emergency patients admitted to hospital at weekends and on weekdays, employing a difference-in-difference analysis [15] to minimise bias from between-Trust differences in case mix and staffing. Conceptually, seven-day services is the research domain, and 'HiSLAC implementation' the research intervention, manifest as an increase in specialist staffing at weekends.

Participating hospital Trusts:

From the 121 acute hospital Trusts participating in HiSLAC, 20 will be invited to participate based on Trust size and Sunday specialist intensity derived from the HiSLAC annual point prevalence survey [4]. Trusts will first be grouped into size quintiles based on the number of beds. Within each quintile, hospitals will be ranked by Sunday specialist intensity (hours per 10 emergency admissions) and two Trusts selected from the top and bottom of the intensity spectrum. In the largest Trust quintile, we will also match on number of acute hospitals within each Trust to ensure balance between single-site and multiple-site Trusts.

Reviewer Recruitment and training

All participating Trusts in England will be invited to self-nominate two registrars (residents) in an acute medical speciality as reviewers, under the indirect supervision of each Trust's HiSLAC local project lead (LPL, a senior consultant (attending)). We anticipate recruiting 80 registrars and 40 LPLs. The reviewers will undertake a training course covering the principles

For peer review only - http://bmjopen.bmj.com/site/about/guidelines.xhtml

of case reviews, providing familiarisation with the scanned case records, and permitting practice with samples of case records allowing feedback and discussion.

Patient case record selection*:*

We will evaluate 4000 non-operative emergency admissions to 20 Trusts to measure variations in quality of care between weekends and weekdays, between high-intensity and low-intensity Trusts, and over time. Case records will be randomly selected from across the whole period of two epochs: financial year 1st April 2012-31st March 2013, and 1st April 2016-31st March 2017, to capture the pre-policy and post-policy intervention periods of specialist staffing respectively, and to avoid bias from seasonal variation. Each epoch will include 100 case records of adult (age 17 years or more) emergency non-operative admissions from each of 20 hospitals, 10 with high and 10 with low-intensity specialist led care (2000 case records for each epoch, 4000 case records in total). Half the records from each centre will be weekend admissions (midnight Friday to midnight Sunday), half weekday. We have chosen non-operative admissions because the surgical population is currently the subject of a separate national multifaceted quality improvement programme, EPOCH (http://www.epochtrial.org/). The analysis is not restricted to mortality reviews, as it is important to avoid endogenous selection bias (from the outcome influencing the sample). Moreover, deaths are not representative of the general in-patient population [16], and avoidable deaths formed only 3.6% of 3400 records in a recent review [17] (less than 1% when adjusting for inconsistency between reviewers [18]).

Case Record Processing (Fig 1):

Patient records will be selected randomly at the University of Birmingham (UoB) from Patient Administration System (PAS) patient records which will have been anonymised at each collaborating Trust, allocated an encrypted code, and transmitted by secure file transfer protocol to be stored on a secure server at UoB. As case records may be missing, Trusts will be provided with 260 codes of which 65 will be weekend and 65 weekday admissions for each epoch; record retrieval will stop once 50 have been obtained in each group (200 total). Following case selection, the encrypted code will be sent to the Trust medical records department, converted to the local NHS number, and the records retrieved for masking to remove patient identifiers. Only the first seven days following admission will be copied, as the purpose of the review is to focus on care around the time of admission, not the totality of the patient's course in hospital. The redacted records will then be scanned as pdf documents, and transmitted by secure file transfer protocol to UoB (Fig 1). Thereby, case records will be anonymised and digitised before being presented to the reviewers.

The Review Process (Fig 2 and Appendix):

We will employ retrospective structured implicit case record review (SICRR) to detect errors in care [17, 19, 20], inviting reviewers to produce short narrative judgements with a 5-point Likert scale rating of overall care quality. Reviewers will be asked to identify the location

For peer review only - http://bmjopen.bmj.com/site/about/guidelines.xhtml

and date of any errors, associate them with specific domains of care (e.g: investigation, diagnosis, monitoring, etc), and to make a judgement about whether the errors caused harm to the patient in terms of adverse events.

Four thousand case records will be reviewed (100 from each epoch, 200 total from each of 20 trusts). Each Trust's collection of 200 case records will be divided into three (non-overlapping) sets of 80 records as follows: Set 1 is a random collection of 80 case records from Epoch 1; Set II is a random collection of 80 case records from Epoch 2; and Set III is the remaining 40 case records (20 from each epoch), with each case record duplicated to create 80 records. Each of the 80 reviewers will be randomly allocated just one case record from each of the three sets above, that is, three case records from each Trust. Under this scheme each reviewer performs 60 reviews and each Trust contributes 40 case records to be reviewed twice; inter-rater reliability can be assessed from the 800 (20%) case-notes with independent reviews from different reviewers using the global ratings of care quality.

Data extraction:

Reviewers will access case records on-line. The case notes will be presented in random order so that learning and fatigue cannot bias the results. Reviewers will not be informed whether records relate to weekend or weekday admissions, but dates will not be masked from the copied records because of the need to determine the timing of events. Reviewers will work independently; they may discuss interpretation of case records with the senior clinician when there is uncertainty, but will be asked to refrain from discussing case records with each other: discussion between reviewers does not improve reliability [21]. The large number of reviewers contributing to each epoch will improve 'calibration' i.e.; reduce the effect of any outliers ('hawks' and 'doves'). After completion of 10 reviews we will compare the number of errors identified and global ratings from each of the 80 reviewers to see if there are any extreme hawks or doves.

Data extraction will be performed by the reviewers using a standardised web-based reporting template (Appendix 2, based on prior work by Hogan [17] and others [22, 23], and on the national Mortality Review process [20]). Assessment of quality of care will be censored at seven days following admission, because the primary aim of the analysis is to examine factors around the time of admission, not later in the patient's stay. Reviewers will be asked to note the date (from which elapsed time from admission can be calculated) of any identified errors, so that proximity to the admission point can be recorded. Reviewers will note only those adverse events associated with errors, and assess preventability.

Process and Outcome Metrics

Errors of omission or commission will be defined as '*the failure of a planned action to be completed as intended or use of a wrong, inappropriate, or incorrect plan to achieve an aim*' (World Health Organisation's taxonomy [24]). Reviewers will be asked to categorise the errors they identify, permitting subsequent comparisons of typology of error (Appendix 1).

For peer review only - http://bmjopen.bmj.com/site/about/guidelines.xhtml

Adverse events are defined as '*An event that results in unintended harm to the patient by an act of commission or omission rather than by the underlying disease or condition of the patient'* [24, 25]. Reviewers will grade the preventability of the adverse event using a standard 6-point scale [26]. This approach permits assessment of the consequences of the errors, without asking reviewers to collect information on all adverse events, many of which will have arisen as a recognised complication or consequence of illness rather than from a lapse in clinical care.

Global (implicit) measure of quality: reviewers will provide an overall assessment of care quality, using a 5-point Likert scale in response to the question 'To what extent did this patient receive best practice care?"

Specialist intensity ('HiSLAC Implementation'): Specialist (consultant) direct involvement in patient care will be derived from the annual High-intensity Specialist Led Acute Care (HiSLAC) point prevalence survey [4]. This self-report web-based survey is administered in June each year; all specialists in 121 participating Trusts across England are invited to record whether they were in the hospital on a particular Sunday or the following Wednesday providing direct clinical care to patients admitted as emergencies, and if so, how many hours they spent doing so.

ANALYTICAL PLAN:

Quality of care: We will examine whether weekend-weekday error rates vary by admission epoch and degree of HiSLAC implementation (specialist hours per 10 emergency admissions). We will look for a difference in the difference between weekdays and weekends across low and high intensity hospitals, and a further difference between epochs. In this way each hospital acts as its own control, thereby adjusting for variation in institutional case mix. We hypothesize that Epoch 1 error rates (the proportion of case records with one or more errors) will be in the region of 20%, and that this rate will diminish between epochs. Adverse event rates have been reported consistently to affect around 10% of patient episodes, of which half are reported as being preventable [26]. As we will only document adverse events considered to be associated with an error in care, the incidence of adverse events in our data set is likely to be lower than 10%, but the preventability rate higher.

Error Typology: Error types will be classified by the reviewers using the coding in Appendix 1. In addition we will perform a qualitative comparison of the difference between reviewers in their typology of errors. Reviewers' free text descriptions of error events will be imported into NVivo for further comparative analysis based on a coding frame developed from a sample of transcripts reviewed and discussed by the members of the project team (including clinical and non-clinical members). The focused coding frame will then be applied to the free-text descriptors to derive a separate classification of error types, permitting a comparison between the a priori classification (derived from Hogan et al [17]) and a grounded approach.

For peer review only - http://bmjopen.bmj.com/site/about/guidelines.xhtml

Health economics: We will use these data to inform the development of the Bayesian model proposed in our parallel health economics modelling [14]. The parameter estimates obtained for error rates from the difference in difference in difference type of analysis will be used to update a prior probability distribution obtained from experts. The elicitation of these prior probabilities will be informed by data from the HiSLAC mixed-methods systematic review of the weekend effect [12] and information derived from the parallel HiSLAC ethnographic study of the 20 hospitals [13]. The methodology for elicitation of this 'prior' and for the updating process has been described elsewhere [14]. In order to calculate the cost utility/benefit of the 7-day services policy of increasing specialist intensity, three further parameters are needed: a) the error rates must be converted into adverse event rates and hence into QALYs using the method of Yao et al [27] and Lilford et al [28]; b) potential cost savings from (any) reduction in adverse events will be calculated, again according to Yao et al [27]; and c) the cost of the intervention itself will be calculated building on Meacock et al [29]. Net costs will be derived by subtracting cost (b) from cost (c). The intervention will dominate if these are negative (since such a scenario can only arise if there is a substantial decrease in adverse events).

Statistical Analysis

Rates of errors and adverse events will be analysed using mixed effects logistic regression models; and the quality of care Likert scale analysed using mixed effects ordinal logistic models, supplemented by linear analyses of the ordinal values. The models will incorporate additive random effects for case-notes, reviewers and hospital trusts and fixed effects for day of week (weekend/weekday), time epoch and their interaction. Trust-level weekend effects will be extracted from these analyses. Changes in trust-level effects between epochs will be correlated with contemporaneous changes in specialist involvement estimated from the point prevalence survey.

Reviewer reliability coefficients will be computed as the proportion of the total variance due to variation between case-notes.

Power and detectable differences

The balanced nature of the design ensures that reviewer and Trust effects are eliminated from the comparisons of interest, at least to a good approximation. Based on earlier work [18] we may assume that 30% of the remaining variance will be attributable to variation between case-notes. From Hutchison et al [19], the base error rate is taken as 20% and the standard deviation of the Likert global quality scale estimated to be 1.06 (this value is adjusted to take account of the smaller number of Likert categories used in the current study – i.e. 5 instead of 6).

Using a P-value of 5%, the following effects will be detectable with 80% power: (a) a difference in weekend/weekday error rates of 3.2% (from 20 trusts, both epochs); (b) a change over time in the weekend/weekday difference of 6.4%; (c) an average difference between weekend and weekday Likert care scores of 0.08 (which may be interpreted as a

For peer review only - http://bmjopen.bmj.com/site/about/guidelines.xhtml

shift of one Likert category for 1 case in every 12; and (d) a change over time in this difference of 0.17.

DISCUSSION

This protocol describes using retrospective case record review to determine whether the quality of care received by patients admitted to hospital as emergencies, varies between weekdays and weekends. The study will contribute to our understanding of the 'weekend effect' and the implementation of seven-day services policy by integrating data on error rates and adverse events with parallel workstreams on specialist staffing [4], a systematic review and framework synthesis of the weekend effect [12], ethnographic evaluations [13], and Bayesian health economics modelling [14].

The strengths of this novel approach lie in triangulating multiple sources of information on the implementation of national health policy, and the use of difference-in-difference analyses to minimise bias from variations in institutional structures and case mix.

Case record review is an imperfect instrument for measuring quality of care. Conventional approaches include explicit criterion-based methods (framed by a checklist) or implicit judgement-based methods. While both are widely used, there are important differences between them. Criterion-based review generally provides a higher level of inter-rater agreement than implicit review, but has been criticised for its insensitivity to detecting more nuanced aspects of care quality, and because the criteria themselves may be susceptible to selection bias. The implicit approach allows reviewers to exercise expert clinical judgement in identifying determinants of quality which may have been omitted from pre-defined criteria, such as evaluating accuracy of diagnosis and appropriateness of care pathways, but this requires a certain level of clinical experience. Midway between these two approaches is the structured implicit case record review (SICRR) [17, 19, 20], which is the method we will adopt here. Recommended for the national mortality review process supported by the Royal College of Physicians [20], SICRR invites reviewers to produce short narrative judgements of care quality in specific phases of care, with a 6-point rating of care quality. The case record review process follows that adopted by Benning et al [30], using explicit (criterion based) and implicit (holistic) approaches since they identify a different spectrum of errors [31, 32]. Implicit review is essential to this study since specialist care is most likely to impact on selecting the correct clinical pathway through accurate diagnosis rather than adhering to that pathway once identified, which is where explicit review has its focus.

Our primary measure of quality will be error rates. We have chosen to focus on processes of care rather than outcomes (adverse events) because process assessment provides a broader foundation on which to assess safety and quality of care, whereas adverse events may arise from the natural history of the disease [16, 31, 32, 33]. We recognise that reliability between reviewers may be higher for diseases with a strong evidence base [34] and weaker for the likely diverse set of conditions associated with emergency admission to hospital as in this review. To mitigate this effect reviewers will be asked to categorise the errors they identify, permitting subsequent comparisons of typology of error (Appendix 1).

For peer review only - http://bmjopen.bmj.com/site/about/guidelines.xhtml

Reviewers will also be asked to document whether or not the errors they identify were associated with an adverse event, thereby linking process to outcome [35], and to grade the preventability of the adverse event using a standard 6-point scale [27]. This permits assessment of the consequences of the errors, without asking reviewers to collect information on all adverse events regardless of causation.

We will not restrict the analysis to mortality reviews, because although adverse events (AEs) and preventable AEs are twice as frequent amongst hospital non-survivors as survivors, and error rates might therefore also be over-represented in this population, these are not representative of the general in-patient population [16]. Random selection of case records from all non-operative admissions will minimise risk of bias which might be associated with random selection of deaths, as the intervention may change the risk of the outcome which would also change the chance of being in the sample (endogenous selection).

ETHICS AND DISSEMINATION

The HiSLAC study, including the case record reviews, was approved by the Review Sub-committee of the South West Wales REC on 11 November 2013. Informed consent was not required for accessing anonymised patient case records from which patient identifiers had been removed. The findings of this study will be published in peer-reviewed journals, the outputs from this research will also form part of the project report to the HS&DR Programme Board.

Competing interests

JB is the Chair, NICE Acute Medical Emergencies Guideline Development Group National Clinical Guideline Centre. All other authors declare no competing interests.

Data sharing statement

We shall make findings available to the scientific community with as few restrictions as feasible when the study has been completed.

Author Contributions

JB and RL developed the initial study idea. CA, JB and AB obtained statutory and ethics approval. JB, CA, AG, GR, CB, TE, MT, CR, MC, AB, CT, ES, JS, PR, RM, YC, SW and RL informed the protocol methodology. All authors contributed to writing the final report and approved the final version.

For peer review only - http://bmjopen.bmj.com/site/about/guidelines.xhtml

REFERENCES

1. Bell CM, Redelmeier DA. Mortality among patients admitted to hospitals on weekends as compared with weekdays. N Engl J Med. 2001 Aug 30;345(9):663-8.
2. Hansard, 13th Oct 2015. https://www.publications.parliament.uk/pa/cm201516/cmhansrd/cm151013/debtext/151013-0001.htm
3. NHS England 2013. https://www.england.nhs.uk/ourwork/qual-clin-lead/seven-day-hospital-services/the-clinical-case/
4. Aldridge C et al. Weekend specialist intensity and admission mortality in acute hospital trusts in England: a cross-sectional study. The Lancet 388.10040 (2016): 178-186.
5. Meacock R, Anselmi L, Kristensen SR, Doran T, Sutton M. Higher mortality rates amongst emergency patients admitted to hospital at weekends reflect a lower probability of admission. J Health Serv Res Policy 2016; May 6. pii: 1355819616649630.
6. Mikulich O, Callaly E, Bennett K, O'Riordan D, Silke B. The increased mortality associated with a weekend emergency admission is due to increased illness severity and altered case-mix. Acute Med. 2011;10(4):182-7.
7. Vest-Hansen B, Riis AH, Sørensen HT, Christiansen CF. Out-of-hours and weekend admissions to Danish medical departments: admission rates and 30-day mortality for 20 common medical conditions. BMJ Open 2015 Mar 11;5(3):e006731.
8. Goodman EK, Reilly AF, Fisher BT, Fitzgerald J, Li Y, Seif AE, Huang YS, Bagatell R, Aplenc R. Association of weekend admission with hospital length of stay, time to chemotherapy, and risk for respiratory failure in pediatric patients with newly diagnosed leukemia at freestanding US children's hospitals. JAMA Pediatr 2014;168(10):925-31.
9. Anselmi L, Meacock R, Kristensen SR, Doran T, Sutton M. Arrival by ambulance explains variation in mortality by time of admission: retrospective study of admissions to hospital following emergency department attendance in England. BMJ Qual Saf Published Online First: 18 October 2016. doi: 10.1136/bmjqs-2016-005680
10. Sayers A, Whitehouse MR, Berstock JR, Harding KA, Kelly MB, Chesser TJ. The association between the day of the week of milestones in the care pathway of patients with hip fracture and 30-day mortality: findings from a prospective national registry – The National Hip Fracture Database of England and Wales. BMC Medicine 2017; 15:62.
11. Buckley D, Bulger D. Trends and Weekly and Seasonal Cycles in the Rate of Errors in the Clinical Management of Hospitalized Patients. Chronobiology International 2012; 29:7, 947-954
12. Chen YF, Boyal A, Sutton E, Armoiry X, Watson S, Bion J, Tarrant C; HiSLAC Collaborative. The magnitude and mechanisms of the weekend effect in hospital admissions: A protocol for a mixed methods review incorporating a systematic review and framework synthesis. Syst Rev. 2016 May 21;5(1):84.
13. Tarrant C, Sutton E, Angell E, Aldridge CP, Boyal A, Bion J on behalf of the HiSLAC study. The ‘weekend effect’ in acute medicine: a protocol for a team-based ethnography of weekend care for medical patients in acute hospital settings. BMJ Open 2017;7:e016755. doi: 10.1136/bmjopen-2017-016755

For peer review only - http://bmjopen.bmj.com/site/about/guidelines.xhtml

14. Watson S et al – Protocol for the health economic evaluation of increasing the weekend specialist to patient ratio submitted 2017 BMJ Open.
15. Sutton M, Nikolova S, Boaden R, Lester H, McDonald R, Roland M. Reduced mortality with hospital pay for performance in England. N Engl J Med. 2012 Nov 8;367(19):1821-8.
16. Baines RJ, Langelaan M, de Bruijne MC, Wagner C. Is researching adverse events in hospital deaths a good way to describe patient safety in hospitals: a retrospective patient record review study. BMJ Open 2015 Jul 9;5(7):e007380. doi: 10.1136/bmjopen-2014-007380.
17. Hogan H, Zipfel R, Neuburger J, Hutchings A, Darzi A, Black N. Avoidability of hospital deaths and association with hospital-wide mortality ratios: retrospective case record review and regression analysis. BMJ. 2015 Jul 14;351:h3239. doi: 10.1136/bmj.h3239.
18. Manaseki-Holland S, Lilford RJ, Bishop JRB, et al. Reviewing deaths in British and US hospitals: a study of two scales for assessing preventability. BMJ Qual Saf Published Online First: 22 June 2016. doi: 10.1136/bmjqs-2015-004849
19. Hutchinson A, Coster JE, Cooper KL, Pearson M, McIntosh A, Bath PA. A structured judgement method to enhance mortality case note review: development and evaluation. BMJ Qual Saf. 2013 Dec;22(12):1032-40. doi: 10.1136/bmjqs-2013-001839.
20. Royal College of Physicians. Using the structured judgment review method – a clinical governance guide to mortality case record reviews. London: RCP, 2016.
21. Hofer TP, Bernstein SJ, DeMonner S, Hayward RA. Discussion between Reviewers Does Not Improve Reliability of Peer Review of Hospital Quality. Medical Care 2000; 38(2): 152-161
22. Vincent, C., G. Neale, and M. Woloshynowych, Adverse events in British hospitals: preliminary retrospective record review. BMJ, 2001. 322: p. 517-9.
23. Hayward, R. and T. Hofer, Estimating Hospital Deaths Due to Medical Error-Preventability is in the eye of the reviewer. JAMA, 2001. 286(4): p. 415-20.
24. WHO 2009. Final Technical Report for The Conceptual Framework for the International Classification for Patient Safety v.11. Technical Annex 2 - Glossary of Patient Safety Concepts and References. www.who.int/patientsafety/taxonomy/icps_technical_annex2.pdf
25. Aspden P, Corrigan J, Wolcott J, Erickson S, editors. Institute of Medicine, Committee on Data Standards for Patient Safety, Board on Health Care Services. Patient Safety: Achieving a New Standard for Care. Washington DC: National Academies of Sciences, 2004.
26. Mayor S, Baines E, Vincent C, Lankshear A, Edwards A, Aylward M, et al. Measuring harm and informing quality improvement in the Welsh NHS: the longitudinal Welsh national adverse events study. Health Serv Deliv Res 2017;5(9)
27. Yao GL, Novielli N, Manaseki-Holland S, Chen Y-F, van der Klink M, Barach P, Chilton PJ, Lilford RJ. Evaluation of a predevelopment service delivery intervention: an application to improve clinical handovers. BMJ Qual Saf 2012; 21 Suppl 1: i29-38.
28. Lilford RJ, Girling AJ, Sheikh A, Coleman JJ, Chilton PJ, Burn SL, Jenkinson DJ, Blake L, Hemming K. Protocol for evaluation of the cost-effectiveness of ePrescribing systems and candidate prototype for other related health information technologies. BMC Health Serv Res 2014; 14(1): 314.

For peer review only - http://bmjopen.bmj.com/site/about/guidelines.xhtml

29. Meacock R, Doran T, Sutton M. What are the Costs and Benefits of Providing Comprehensive Seven-day Services for Emergency Hospital Admissions? Health Econ. 2015 Aug;24(8):907-12. Doi: 10.1002/hec.3207. Epub 2015 May 22.
30. Benning A, Ghaleb M, Suokas A, Dixon-Woods M, Dawson J, Barber N, Franklin BD, Girling A, Hemming K, Carmalt M, Rudge G, Naicker T, Nwulu U, Choudhury S, Lilford R. Large scale organisational intervention to improve patient safety in four UK hospitals: mixed method evaluation. BMJ. 2011 Feb 3;342:d195. doi: 10.1136/bmj.d195.
31. Lilford RJ, Brown CA, Nicholl J. Use of process measures to monitor the quality of clinical practice. BMJ. 2007 Sep 29;335(7621):648-50.
32. Lilford RJ, Mohammed MA, Braunholtz D, Hofer TP. The measurement of active errors: methodological issues. Qual Saf Health Care. 2003 Dec;12 Suppl 2:ii8-12.
33. Hofer TP. Commentary on Sharek: adverse events and errors-important to differentiate and difficult to measure. Health Serv Res. 2011 Apr;46(2):679-84.
34. Hofer TP, Asch SM, Hayward RA, Rubenstein LV, Hogan MM, Adams J, Kerr EA. Profiling quality of care: Is there a role for peer review? BMC Health Serv Res. 2004 May 19;4(1):9.
35. National Patient Safety Agency. Seven Steps to Patient Safety - your guide to safer patient care. NPSA London 2004

For peer review only

For peer review only - http://bmjopen.bmj.com/site/about/guidelines.xhtml

FIGURES

Fig 1: Case record processing

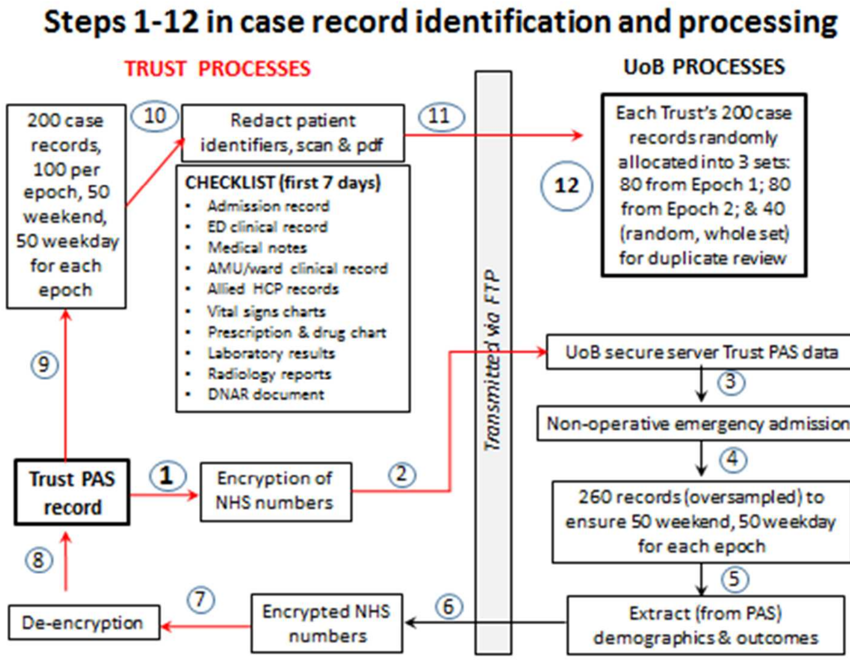

Fig 2: The Review Process

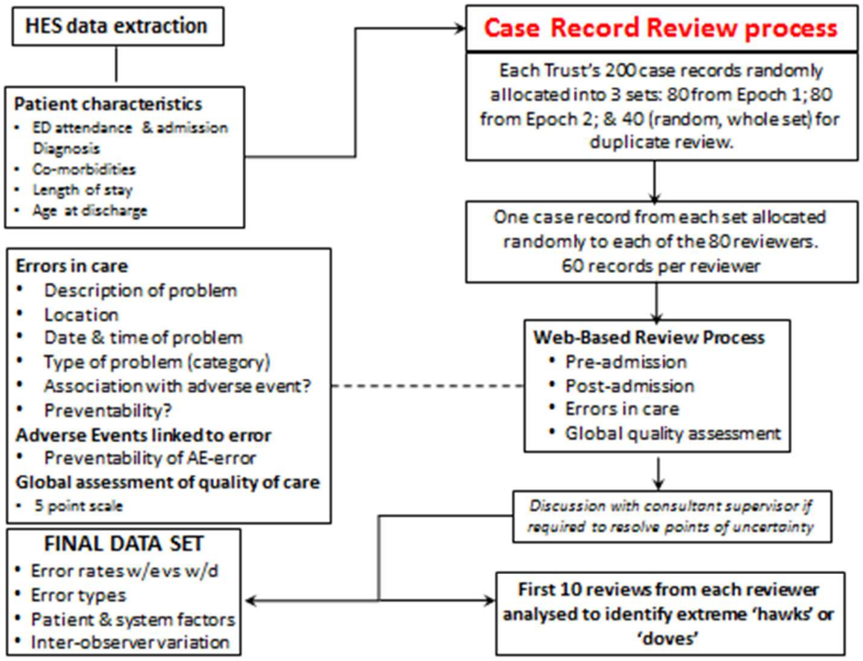

APPENDICES

For peer review only - http://bmjopen.bmj.com/site/about/guidelines.xhtml

APPENDIX 1: REFERENCE MATERIALS

**1.1 Location Of Error: Where did the error occur?**
A. Outside hospital (primary care, ambulance, etc.)
B. In the ED or linked area before admission
C. AMU or equivalent area
D. Acute ward (other than AMU)
E. Speciality ward (ICU/HDU, coronary care, renal, respiratory, elderly care, rehab)
F. Diagnostic area, radiology

**1.2 Error Typology**	***Examples only (not exhaustive – not for recording)***
A. Assessment, investigation or diagnosis	Physical examination and history taking incomplete<br>Pressure ulcer risk not assessed/incorrectly assessed<br>VTE risk assessment not completed/incorrectly completed<br>Falls history/vulnerability to falls not identified<br>Swallowing safety not assessed/incorrectly assessed<br>Tests and investigations missed/delayed/wrong<br>Diagnosis missed/delayed/wrong<br>Failure to assess comorbidities or frailty
B. Medication	Over- or under-hydration<br>Oxygen supply wrong/delayed/omitted<br>Allergic/anaphylactic reaction to any medication<br>Anticoagulants/antiplatelets wrong/delayed/omitted<br>Antibiotics wrong/delayed/omitted<br>Insulin or other diabetes medication wrong/delayed/omitted<br>Opiates wrong/delayed/omitted<br>Sedatives/hypnotics/antipsychotics wrong/delayed/omitted<br>Steroids wrong/delayed/omitted<br>NSAID wrong/delayed/omitted<br>Diuretics wrong/delayed/omitted<br>Antihypertensives wrong/delayed/omitted<br>Cardiovascular medications wrong/delayed/omitted<br>Chemotherapy wrong/delayed/omitted
C. Treatment and management plan	Appropriate medical/surgical treatment not planned<br>Avoidable delay/omission of planned medical/surgical treatment<br>Inappropriate/unnecessary medical/surgical treatment given<br>Inappropriate ceiling of care<br>Omitted/delayed/wrong treatment from AHPs<br>Acquired pressure ulcer: prevention below acceptable standard<br>Acquired pressure ulcer despite apparently acceptable standard of prevention<br>Slip/trip/fall: prevention plan below acceptable standard

For peer review only - http://bmjopen.bmj.com/site/about/guidelines.xhtml

	Slip/trip/fall despite apparently acceptable standard of falls prevention<br>Developed VTE: prophylaxis below acceptable standard<br>Developed VTE despite apparently acceptable standard of VTE prophylaxis
D. Infection control	Surgical wound infection<br>Infection from invasive procedure other than surgery<br>Other healthcare associated wound infection *(e.g. infected ulcer)*<br>Infection from indwelling device *(catheter, central lines, etc.)*<br>Healthcare associated clostridium difficile<br>Healthcare- /device-associated MRSA bloodstream infection<br>Other bloodstream infection (not MRSA)<br>Healthcare associated pneumonia/chest infection (including aspiration)<br>Healthcare associated norovirus/D&V
E. Invasive procedures	Avoidable delay in undertaking procedure<br>Inadequate pre-procedure assessment/preparation<br>Complication of anaesthesia/sedation including airway management<br>Complication of operative procedure *(e.g. perforation, haemorrhage)*<br>Complication of invasive procedure *(e.g. perforation, haemorrhage)*
F. Monitoring	Vital signs monitoring.<br>Fluid intake/output.<br>Nutritional intake.<br>Delay in initiating resuscitation
G. Cardiopulmonary resuscitation	Delay in initiating cardiopulmonary resuscitation<br>Inappropriate resuscitation<br>Airway management<br>Resuscitation equipment
H. Other problem	Describe in free text

**1.3: Grading Of Preventability Of Adverse Event:**
1. Virtually no evidence for preventability.
2. Slight to modest evidence of preventability.
3. Possibly preventable, but not very likely (less than 50–50, but close call).
4. Probably preventable (more than 50–50, but close call).

For peer review only

For peer review only - http://bmjopen.bmj.com/site/about/guidelines.xhtml

5. Strong evidence for preventability
6. Virtually certain evidence of preventability.

**1.4: Global assessment of quality of care**
To what extent did this patient receive best practice care? (select one only)
• Completely
• Substantially
• Partially
• Very little
• Not at all

For peer review only

For peer review only - http://bmjopen.bmj.com/site/about/guidelines.xhtml

Appendix 2: HiSLAC Case Record Review Data Collection

On registration of each reviewer, we will allocate an unique Reviewer code which will function as log-in user name and link to the details below:

Name:	
Email:	
Phone number:	
Date of Birth (age)	
Gender	
Grade: Specialist Registrar training years 5-7, or Consultant	
Year of graduation:	
Current speciality/specialities (CCT programmes) –drop-down list	

TO BE RECORDED AUTOMATICALLY AT THE BEGINNING OF EACH CASE RECORD REVIEW

- Patient unique study number:
- Reviewer's Code
- Date and time of the review:

DATA COLLECTION

1. **HES DATA EXTRACTED BY PROJECT TEAM BEFORE CASE RECORD REVIEW (not made available to reviewers)**	
Sex M/F	
Age at hospital admission	
Date and Time of patient's first arrival at hospital (ED or other primary receiving ward).	
Duration of stay in ED (interval between arrival at hospital and time of admission, hrs & mins)	
Time, date and day of admission	
Length of hospital stay (days)	
Primary admitting diagnosis	
Comorbid disease (Charlson)	
Hospital Outcome: death or survival	

**2. CLINICAL DATA EXTRACTED BY CASE RECORD REVIEWER (independently for duplicate reviews)**	
1. **PRE-ADMISSION PHASE INCLUDING EMERGENCY DEPARTMENT**	
Source of admission:<br>• Own home<br>• Nursing or residential care home<br>• Another hospital<br>• No fixed abode<br>• No information available	
Patient condition immediately before the illness that led to this admission.<br>• Independent<br>• Needing help with some activities of daily living (ADLs)	

For peer review only

For peer review only - http://bmjopen.bmj.com/site/about/guidelines.xhtml

• Dependant on others for most/all ADLs including personal hygiene<br>• Unable to determine; no relevant information in notes	
Referral mechanism:<br>• Self-presentation to ED (walk-in/own transport)<br>• 999/ambulance transfer to ED<br>• GP or deputising service referral<br>• Unable to determine	
Admission pathway:<br>Was the patient initially assessed in ED or any other short term emergency pre-admission assessment unit (e.g. Clinical Decision Unit, Ambulatory care, Medical or Surgical Assessment Unit, etc.), or was the patient admitted directly to an acute ward (AMU, general or specialty ward)?<br>• ED/pre-admission area<br>• Direct admission to acute ward<br>• Unable to determine	
2. **POST-ADMISSION PHASE**	
Location immediately following admission:<br>• Clinical Decision Unit or short stay ward<br>• Acute Medical Unit (AMU/MAU)<br>• General medical ward<br>• Medical sub-specialities including high care (eg: Coronary Care Unit, Renal Unit, Respiratory, Haematology, Oncology)<br>• Older People's Medicine/Elderly Care Unit<br>• Rehabilitation<br>• Critical Care Unit / Intensive Care Unit (including High Dependency)<br>• General Surgery (including surgical assessment/operating theatre)<br>• Other (please specify):<br>• Unable to determine ward type	
Was this an appropriate type of ward for the patient's condition?<br>• Yes, definitely appropriate<br>• Probably appropriate<br>• No<br>• Unable to determine	
Were vital signs recorded for calculation of a National Early Warning Score in the first 12 hours following admission?<br>• Yes, full vital signs and a NEWS recorded<br>• Full vital signs, but NEWS not documented<br>• Some vital signs not documented, no NEWS<br>• No evidence of vital signs or NEWS	
Initial Consultant Review in the first 24 hours following admission:<br>• Consultant review documented [REVIEWER TO RECORD TIME AND DATE]<br>• Probable consultant review but status of doctor uncertain [REVIEWER TO RECORD TIME AND DATE]<br>• Consultant review, time not documented, but case record suggests < 14 hrs after admission<br>• Consultant review, time not documented, but case record suggests > 14 hrs after admission<br>• Unlikely that consultant review occurred during first 24 hours<br>• No evidence for consultant review in first 24 hours	

For peer review only

For peer review only - http://bmjopen.bmj.com/site/about/guidelines.xhtml

Palliative and end-of-life care (within first 7 days): were discussions held or decisions made to limit treatment, forego resuscitation (DNACPR), or refer to palliative care?<br>• No: not required, patient appropriately for full treatment<br>• No, but would probably have been appropriate to consider some form of treatment limitation<br>• No, but would definitely have been appropriate to limit treatment.<br>• Yes: time and date of discussion or decision to limit treatment<br>○ Yes, appropriate decision<br>○ Yes, but patient might have benefited from escalation<br>○ Yes, but likely inappropriate decision, patient should have been considered for full escalation<br>• If referral to palliative care, time and date referral made:	
Admission avoidance: Could admission to hospital have been avoided given optimal care in the community (eg: primary care, Hospital at Home, palliative care etc)?<br>• Yes<br>• Possibly<br>• No	
Free Text Comments: Anything else you want to mention about this case?	
**3. ASSESSMENT OF CARE QUALITY**	
Errors in care:<br>Does the case record contain evidence of one or more errors in care defined as '*the failure of a planned action to be completed as intended or use of a wrong, inappropriate, or incorrect plan to achieve an aim*' (This may include events identified above). If Yes, please complete next table, errors in healthcare:<br>• Description of event<br>• Where did the event occur?<br>• When did the event occur?<br>• Category of event<br>• Associated with adverse event?<br>• If yes, grade preventability	
Proceed to next table, errors in care	

For peer review only - http://bmjopen.bmj.com/site/about/guidelines.xhtml

Errors in care. Please complete the following table using the categories listed below this table

- **An error in care is defined as '**the failure of a planned action to be completed as intended or use of a wrong, inappropriate, or incorrect plan to achieve an aim**'.** Errors may or may not have direct consequences for the patient, but they should have the potential to have adverse consequences. To identify errors**,** consider what an acceptable standard of healthcare would be for this patient, and describe whether (and how) the healthcare they received fell below this acceptable standard (whether through commission, omission, delay or incorrect actions). **Only one error should be entered per row.**
- **Where did the error occur (Appendix 1.1)?**
- **When did the error occur (enter date and time)**
- **What was the category of error (Appendix 1.2)? More than one may be chosen, in which case please list the most important first.**
- **Was the error associated with an adverse event (AE)? We define an AE as '***An event that results in unintended harm to the patient by an act of commission or omission rather than by the underlying disease or condition of the patient'***. Responses: Yes / No / Insufficient evidence**
- **Preventability: If in your opinion the error resulted in an adverse event, to what extent was the adverse event preventable? Please use the grading below.**
  1. Virtually no evidence for preventability.
  2. Slight to modest evidence of preventability.
  3. Possibly preventable, but not very likely (less than 50–50, but close call).
  4. Probably preventable (more than 50–50, but close call).
  5. Strong evidence for preventability
  6. Virtually certain evidence of preventability.

For peer review only - http://bmjopen.bmj.com/site/about/guidelines.xhtml

**Describe each error in your own words. Please describe what should have happened AND what did happen.**	**Where did the error occur? (*Appendix 1.1*)**	**When did the error occur? (*Date, time*)**	**Category of error (*Appendix 1.2*)**	**Was the error associated with an adverse event? (*Y / N / insufficient evidence*)**	**To what extent was the adverse event preventable (*Appendix 1.3, grade 1-6*)?**
Example: "First dose of IV antibiotics should have been given immediately but was not given until three hours after prescribed"	*B*	*Date and time*	*B*	*N*	*n/a*
Example: Inappropriate administration of thrombolysis for an assumed minor pulmonary embolism, when in fact the patient had sustained a pulmonary haemorrhage.	*C*	*Date & time*	*A & B*	*Y*	*4*
Example: no vital signs / NEWS records were documented in the first 12 hours following transfer from the AMU to the ward. The patient suffered a cardiac arrest from which she could not be resuscitated.	*D*	*Date & time*	*F*	*Y*	*5*
Example: a single dose of a NSAID was given to this patient with AKI; the prescription was cancelled by the consultant the following morning.	*D*	*Date & time*	*B*	*N*	*n/a*

For peer review only - http://bmjopen.bmj.com/site/about/guidelines.xhtml

Describe each error in your own words. Please describe what should have happened AND what did happen.	Where did the error occur? (*Appendix 1.1*)	When did the error occur? (*Date, time*)	Category of error (*Appendix 1.2*)	Was the error associated with an adverse event? (*Y / N / insufficient evidence*)	To what extent was the adverse event preventable (*Appendix 1.3, grade 1-6*)?

Global assessment of quality of care
To what extent did this patient receive best practice care? (select one only)
• Completely
• Substantially
• Partially
• Very little
• Not at all

How confident are you in your judgement of global quality? (select one only)
☐ Completely confident
☐ Substantially confident
☐ Partially confident
☐ Slightly confident
☐ Not at all confident

For peer review only - http://bmjopen.bmj.com/site/about/guidelines.xhtml

To what extent was the adverse event preventable?
1. Virtually no evidence for preventability.
2. Slight to modest evidence of preventability
3. Possibly preventable, but not very likely (less than 50–50, but close call)
4. Probably preventable (more than 50–50, but close call)
5. Strong evidence for preventability
6. Virtually certain evidence of preventability
n/a = not applicable, no adverse event

For peer review only

For peer review only - http://bmjopen.bmj.com/site/about/guidelines.xhtml

Figures

Fig 1: Case record processing

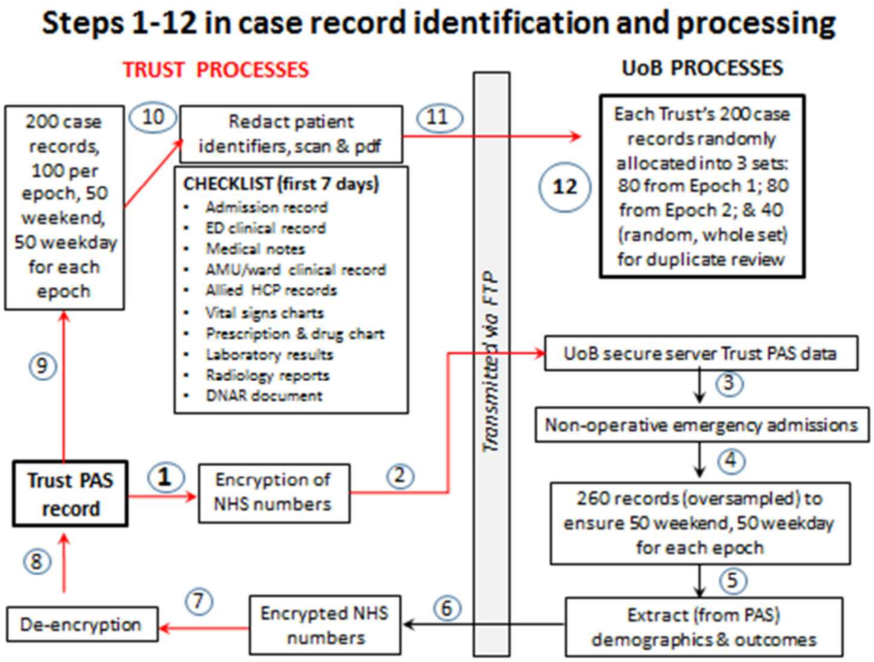

Fig 2: The Review Process

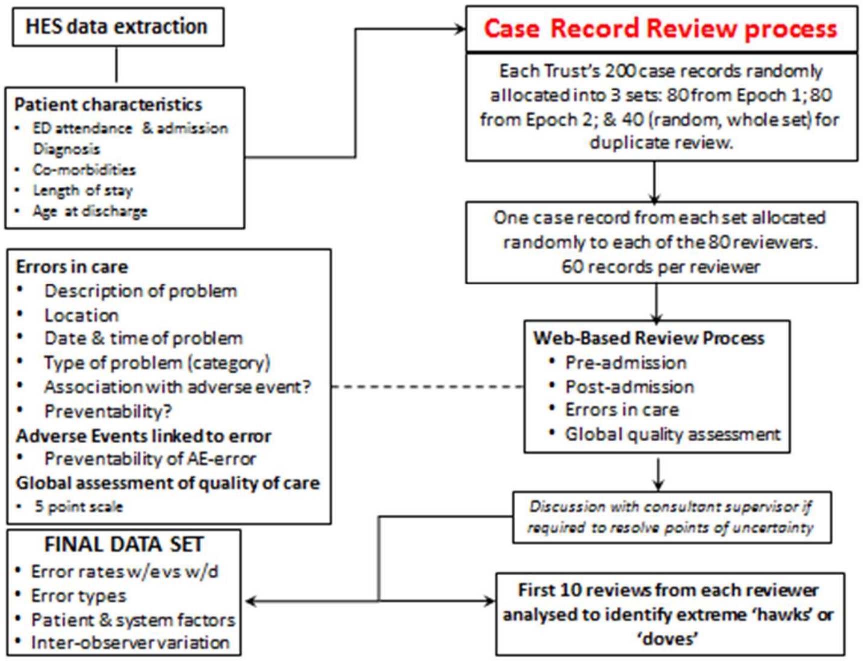

APPENDICES

APPENDIX 1: REFERENCE MATERIALS

**1.1 Location Of Error: Where did the error occur?**
A. Outside hospital (primary care, ambulance, etc.)
B. In the ED or linked area before admission
C. AMU or equivalent area
D. Acute ward (other than AMU)
E. Speciality ward (ICU/HDU, coronary care, renal, respiratory, elderly care, rehab)
F. Diagnostic area, radiology

**1.2 Error Typology**	***Examples only (not exhaustive – not for recording)***
A. Assessment, investigation or diagnosis	Physical examination and history taking incomplete<br>Pressure ulcer risk not assessed/incorrectly assessed<br>VTE risk assessment not completed/incorrectly completed<br>Falls history/vulnerability to falls not identified<br>Swallowing safety not assessed/incorrectly assessed<br>Tests and investigations missed/delayed/wrong<br>Diagnosis missed/delayed/wrong<br>Failure to assess comorbidities or frailty
B. Medication	Over- or under-hydration<br>Oxygen supply wrong/delayed/omitted<br>Allergic/anaphylactic reaction to any medication<br>Anticoagulants/antiplatelets wrong/delayed/omitted<br>Antibiotics wrong/delayed/omitted<br>Insulin or other diabetes medication wrong/delayed/omitted<br>Opiates wrong/delayed/omitted<br>Sedatives/hypnotics/antipsychotics wrong/delayed/omitted<br>Steroids wrong/delayed/omitted<br>NSAID wrong/delayed/omitted<br>Diuretics wrong/delayed/omitted<br>Antihypertensives wrong/delayed/omitted<br>Cardiovascular medications wrong/delayed/omitted<br>Chemotherapy wrong/delayed/omitted

For peer review only

C. Treatment and management plan	Appropriate medical/surgical treatment not planned<br>Avoidable delay/omission of planned medical/surgical treatment<br>Inappropriate/unnecessary medical/surgical treatment given<br>Inappropriate ceiling of care<br>Omitted/delayed/wrong treatment from AHPs<br>Acquired pressure ulcer: prevention below acceptable standard<br>Acquired pressure ulcer despite apparently acceptable standard of prevention<br>Slip/trip/fall: prevention plan below acceptable standard<br>Slip/trip/fall despite apparently acceptable standard of falls prevention<br>Developed VTE: prophylaxis below acceptable standard<br>Developed VTE despite apparently acceptable standard of VTE prophylaxis
D. Infection control	Surgical wound infection<br>Infection from invasive procedure other than surgery<br>Other healthcare associated wound infection *(e.g. infected ulcer)*<br>Infection from indwelling device *(catheter, central lines, etc.)*<br>Healthcare associated clostridium difficile<br>Healthcare- /device-associated MRSA bloodstream infection<br>Other bloodstream infection (not MRSA)<br>Healthcare associated pneumonia/chest infection (including aspiration)<br>Healthcare associated norovirus/D&V
E. Invasive procedures	Avoidable delay in undertaking procedure<br>Inadequate pre-procedure assessment/preparation<br>Complication of anaesthesia/sedation including airway management<br>Complication of operative procedure *(e.g. perforation, haemorrhage)*<br>Complication of invasive procedure *(e.g. perforation, haemorrhage)*
F. Monitoring	Vital signs monitoring.<br>Fluid intake/output.<br>Nutritional intake.<br>Delay in initiating resuscitation

For peer review only

G. Cardiopulmonary resuscitation	Delay in initiating cardiopulmonary resuscitation<br>Inappropriate resuscitation<br>Airway management<br>Resuscitation equipment
H. Other problem	Describe in free text

**1.3: Grading Of Preventability Of Adverse Event:**
1. Virtually no evidence for preventability.
2. Slight to modest evidence of preventability.
3. Possibly preventable, but not very likely (less than 50–50, but close call).
4. Probably preventable (more than 50–50, but close call).
5. Strong evidence for preventability
6. Virtually certain evidence of preventability.

**1.4: Global assessment of quality of care**
To what extent did this patient receive best practice care? (select one only)
• Completely
• Substantially
• Partially
• Very little
• Not at all

Appendix 2: HiSLAC Case Record Review Data Collection

On registration of each reviewer, we will allocate an unique Reviewer code which will function as log-in user name and link to the details below:

Name:	
Email:	
Phone number:	
Date of Birth (age)	
Gender	
Grade: Specialist Registrar training years 5-7, or Consultant	

Year of graduation:
Current speciality/specialities (CCT programmes) –drop-down list

TO BE RECORDED AUTOMATICALLY AT THE BEGINNING OF EACH CASE RECORD REVIEW

- Patient unique study number:
- Reviewer's Code
- Date and time of the review:

DATA COLLECTION

**1. HES DATA EXTRACTED BY PROJECT TEAM BEFORE CASE RECORD REVIEW (not made available to reviewers)**	
Sex M/F	
Age at hospital admission	
Date and Time of patient's first arrival at hospital (ED or other primary receiving ward).	
Duration of stay in ED (interval between arrival at hospital and time of admission, hrs & mins)	
Time, date and day of admission	
Length of hospital stay (days)	
Primary admitting diagnosis	
Comorbid disease (Charlson)	
Hospital Outcome: death or survival	

**2. CLINICAL DATA EXTRACTED BY CASE RECORD REVIEWER (independently for duplicate reviews)**	
1. **PRE-ADMISSION PHASE INCLUDING EMERGENCY DEPARTMENT**	
Source of admission:<br>• Own home<br>• Nursing or residential care home<br>• Another hospital<br>• No fixed abode<br>• No information available	

For peer review only

Patient condition immediately before the illness that led to this admission.<br>• Independent<br>• Needing help with some activities of daily living (ADLs)<br>• Dependant on others for most/all ADLs including personal hygiene<br>• Unable to determine; no relevant information in notes	
Referral mechanism:<br>• Self-presentation to ED (walk-in/own transport)<br>• 999/ambulance transfer to ED<br>• GP or deputising service referral<br>• Unable to determine	
Admission pathway:<br>Was the patient initially assessed in ED or any other short term emergency pre-admission assessment unit (e.g. Clinical Decision Unit, Ambulatory care, Medical or Surgical Assessment Unit, etc.), or was the patient admitted directly to an acute ward (AMU, general or specialty ward)?<br>• ED/pre-admission area<br>• Direct admission to acute ward<br>• Unable to determine	
2. **POST-ADMISSION PHASE**	
Location immediately following admission:<br>• Clinical Decision Unit or short stay ward<br>• Acute Medical Unit (AMU/MAU)<br>• General medical ward<br>• Medical sub-specialities including high care (eg: Coronary Care Unit, Renal Unit, Respiratory, Haematology, Oncology)<br>• Older People's Medicine/Elderly Care Unit<br>• Rehabilitation<br>• Critical Care Unit / Intensive Care Unit (including High Dependency)<br>• General Surgery (including surgical assessment/operating theatre)<br>• Other (please specify):<br>• Unable to determine ward type	
Was this an appropriate type of ward for the patient's condition?<br>• Yes, definitely appropriate<br>• Probably appropriate<br>• No<br>• Unable to determine	
Were vital signs recorded for calculation of a National Early Warning Score in the first 12 hours following admission?<br>• Yes, full vital signs and a NEWS recorded<br>• Full vital signs, but NEWS not documented<br>• Some vital signs not documented, no NEWS<br>• No evidence of vital signs or NEWS	
Initial Consultant Review in the first 24 hours following admission:	

For peer review only

• Consultant review documented [REVIEWER TO RECORD TIME AND DATE]<br>• Probable consultant review but status of doctor uncertain [REVIEWER TO RECORD TIME AND DATE]<br>• Consultant review, time not documented, but case record suggests < 14 hrs after admission<br>• Consultant review, time not documented, but case record suggests > 14 hrs after admission<br>• Unlikely that consultant review occurred during first 24 hours<br>• No evidence for consultant review in first 24 hours	
Palliative and end-of-life care (within first 7 days): were discussions held or decisions made to limit treatment, forego resuscitation (DNACPR), or refer to palliative care?<br>• No: not required, patient appropriately for full treatment<br>• No, but would probably have been appropriate to consider some form of treatment limitation<br>• No, but would definitely have been appropriate to limit treatment.<br>• Yes: time and date of discussion or decision to limit treatment<br>○ Yes, appropriate decision<br>○ Yes, but patient might have benefited from escalation<br>○ Yes, but likely inappropriate decision, patient should have been considered for full escalation<br>• If referral to palliative care, time and date referral made:	
Admission avoidance: Could admission to hospital have been avoided given optimal care in the community (eg: primary care, Hospital at Home, palliative care etc)?<br>• Yes<br>• Possibly<br>• No	
Free Text Comments: Anything else you want to mention about this case?	
**3. ASSESSMENT OF CARE QUALITY**	
Errors in care:<br>Does the case record contain evidence of one or more errors in care defined as '*the failure of a planned action to be completed as intended or use of a wrong, inappropriate, or incorrect plan to achieve an aim*' (This may include events identified above). If Yes, please complete next table, errors in healthcare:<br>• Description of event<br>• Where did the event occur?<br>• When did the event occur?<br>• Category of event	

For peer review only

• Associated with adverse event?<br>• If yes, grade preventability	
Proceed to next table, errors in care	

For peer review only

Errors in care. Please complete the following table using the categories listed below this table

- **An error in care is defined as** 'the failure of a planned action to be completed as intended or use of a wrong, inappropriate, or incorrect plan to achieve an aim'**.** Errors may or may not have direct consequences for the patient, but they should have the potential to have adverse consequences. To identify errors**,** consider what an acceptable standard of healthcare would be for this patient, and describe whether (and how) the healthcare they received fell below this acceptable standard (whether through commission, omission, delay or incorrect actions). **Only one error should be entered per row.**
- **Where did the error occur (Appendix 1.1)?**
- **When did the error occur (enter date and time)**
- **What was the category of error (Appendix 1.2)? More than one may be chosen, in which case please list the most important first.**
- **Was the error associated with an adverse event (AE)? We define an AE as** '*An event that results in unintended harm to the patient by an act of commission or omission rather than by the underlying disease or condition of the patient*'**. Responses: Yes / No / Insufficient evidence**
- **Preventability: If in your opinion the error resulted in an adverse event, to what extent was the adverse event preventable? Please use the grading below.**
  1. Virtually no evidence for preventability.
  2. Slight to modest evidence of preventability.
  3. Possibly preventable, but not very likely (less than 50–50, but close call).
  4. Probably preventable (more than 50–50, but close call).
  5. Strong evidence for preventability
  6. Virtually certain evidence of preventability.

For peer review only

**Describe each error in your own words. Please describe what should have happened AND what did happen.**	**Where did the error occur? (*Appendix 1.1*)**	**When did the error occur? (*Date, time*)**	**Category of error (*Appendix 1.2*)**	**Was the error associated with an adverse event? (*Y / N / insufficient evidence*)**	**To what extent was the adverse event preventable (*Appendix 1.3, grade 1-6*)?**
Example: "First dose of IV antibiotics should have been given immediately but was not given until three hours after prescribed"	*B*	*Date and time*	*B*	*N*	*n/a*
Example: Inappropriate administration of thrombolysis for an assumed minor pulmonary embolism, when in fact the patient had sustained a pulmonary haemorrhage.	*C*	*Date & time*	*A & B*	*Y*	*4*
Example: no vital signs / NEWS records were documented in the first 12 hours following transfer from the AMU to the ward. The patient suffered a cardiac arrest from which she could not be resuscitated.	*D*	*Date & time*	*F*	*Y*	*5*
Example: a single dose of a NSAID was given to this patient with AKI; the prescription was cancelled by the consultant the following morning.	*D*	*Date & time*	*B*	*N*	*n/a*

Describe each error in your own words. Please describe what should have happened AND what did happen.	Where did the error occur? (*Appendix 1.1*)	When did the error occur? (*Date, time*)	Category of error (*Appendix 1.2*)	Was the error associated with an adverse event? (*Y / N / insufficient evidence*)	To what extent was the adverse event preventable (*Appendix 1.3, grade 1-6*)?

Global assessment of quality of care
To what extent did this patient

How confident are you in your judgement of global quality? (select one only)
☐ Completely confident
☐ Substantially confident
☐ Partially confident
☐ Slightly confident
☐ Not at all confident

receive best practice care? (select one only)
• Completely
• Substantially
• Partially
• Very little
• Not at all

**To what extent was the adverse event preventable?**
1. Virtually no evidence for preventability.
2. Slight to modest evidence of preventability
3. Possibly preventable, but not very likely (less than 50–50, but close call)
4. Probably preventable (more than 50–50, but close call)
5. Strong evidence for preventability
6. Virtually certain evidence of preventability
n/a = not applicable, no adverse event

For peer review only

STANDARD PROTOCOL ITEMS: RECOMMENDATIONS FOR INTERVENTIONAL TRIALS

SPIRIT 2013 Checklist: Recommended items to address in a clinical trial protocol and related documents*

Section/item	Item No	Description	Addressed on page number
**Administrative information**			
Title	1	Descriptive title identifying the study design, population, interventions, and, if applicable, trial acronym	Page1 of 37
Trial registration	2a	Trial identifier and registry name. If not yet registered, name of intended registry	See ref below
	2b	All items from the World Health Organization Trial Registration Data Set	______________
Protocol version	3	Date and version identifier	V1
Funding	4	Sources and types of financial, material, and other support	Page 2 of 37
Roles and responsibilities	5a	Names, affiliations, and roles of protocol contributors	Page 1 of 37
	5b	Name and contact information for the trial sponsor	______________
	5c	Role of study sponsor and funders, if any, in study design; collection, management, analysis, and interpretation of data; writing of the report; and the decision to submit the report for publication, including whether they will have ultimate authority over any of these activities	None
	5d	Composition, roles, and responsibilities of the coordinating centre, steering committee, endpoint adjudication committee, data management team, and other individuals or groups overseeing the trial, if applicable (see Item 21a for data monitoring committee)	Page 10 of 37

2a: https://www.journalslibrary.nihr.ac.uk/programmes/hsdr/1212817/#/

For peer review only - http://bmjopen.bmj.com/site/about/guidelines.xhtml

**Introduction**			
Background and rationale	6a	Description of research question and justification for undertaking the trial, including summary of relevant studies (published and unpublished) examining benefits and harms for each intervention	Page 4
	6b	Explanation for choice of comparators	Pages 4 & 5
Objectives	7	Specific objectives or hypotheses	Page 5
Trial design	8	Description of trial design including type of trial (eg, parallel group, crossover, factorial, single group), allocation ratio, and framework (eg, superiority, equivalence, noninferiority, exploratory)	Page 5
**Methods: Participants, interventions, and outcomes**			
Study setting	9	Description of study settings (eg, community clinic, academic hospital) and list of countries where data will be collected. Reference to where list of study sites can be obtained	Page 5
Eligibility criteria	10	Inclusion and exclusion criteria for participants. If applicable, eligibility criteria for study centres and individuals who will perform the interventions (eg, surgeons, psychotherapists)	Page 5
Interventions	11a	Interventions for each group with sufficient detail to allow replication, including how and when they will be administered	Page 5
	11b	Criteria for discontinuing or modifying allocated interventions for a given trial participant (eg, drug dose change in response to harms, participant request, or improving/worsening disease)	Not applicable
	11c	Strategies to improve adherence to intervention protocols, and any procedures for monitoring adherence (eg, drug tablet return, laboratory tests)	Not applicable
	11d	Relevant concomitant care and interventions that are permitted or prohibited during the trial	Not applicable
Outcomes	12	Primary, secondary, and other outcomes, including the specific measurement variable (eg, systolic blood pressure), analysis metric (eg, change from baseline, final value, time to event), method of aggregation (eg, median, proportion), and time point for each outcome. Explanation of the clinical relevance of chosen efficacy and harm outcomes is strongly recommended	Pages 7 & 8
Participant timeline	13	Time schedule of enrolment, interventions (including any run-ins and washouts), assessments, and visits for participants. A schematic diagram is highly recommended (see Figure)	Fig 1&2

For peer review only - http://bmjopen.bmj.com/site/about/guidelines.xhtml

Sample size	14	Estimated number of participants needed to achieve study objectives and how it was determined, including clinical and statistical assumptions supporting any sample size calculations	Page 9
Recruitment	15	Strategies for achieving adequate participant enrolment to reach target sample size	n/a
**Methods: Assignment of interventions (for controlled trials)**			
Allocation:			
Sequence generation	16a	Method of generating the allocation sequence (eg, computer-generated random numbers), and list of any factors for stratification. To reduce predictability of a random sequence, details of any planned restriction (eg, blocking) should be provided in a separate document that is unavailable to those who enrol participants or assign interventions	n/a
Allocation concealment mechanism	16b	Mechanism of implementing the allocation sequence (eg, central telephone; sequentially numbered, opaque, sealed envelopes), describing any steps to conceal the sequence until interventions are assigned	Page 7
Implementation	16c	Who will generate the allocation sequence, who will enrol participants, and who will assign participants to interventions	n/a
Blinding (masking)	17a	Who will be blinded after assignment to interventions (eg, trial participants, care providers, outcome assessors, data analysts), and how	Page 7
	17b	If blinded, circumstances under which unblinding is permissible, and procedure for revealing a participant's allocated intervention during the trial	n/a
**Methods: Data collection, management, and analysis**			
Data collection methods	18a	Plans for assessment and collection of outcome, baseline, and other trial data, including any related processes to promote data quality (eg, duplicate measurements, training of assessors) and a description of study instruments (eg, questionnaires, laboratory tests) along with their reliability and validity, if known. Reference to where data collection forms can be found, if not in the protocol	Pages 6&7
	18b	Plans to promote participant retention and complete follow-up, including list of any outcome data to be collected for participants who discontinue or deviate from intervention protocols	n/a

For peer review only - http://bmjopen.bmj.com/site/about/guidelines.xhtml

Data management	19	Plans for data entry, coding, security, and storage, including any related processes to promote data quality (eg, double data entry; range checks for data values). Reference to where details of data management procedures can be found, if not in the protocol	Fig 1
Statistical methods	20a	Statistical methods for analysing primary and secondary outcomes. Reference to where other details of the statistical analysis plan can be found, if not in the protocol	Page 8
	20b	Methods for any additional analyses (eg, subgroup and adjusted analyses)	Page 8 & 9
	20c	Definition of analysis population relating to protocol non-adherence (eg, as randomised analysis), and any statistical methods to handle missing data (eg, multiple imputation)	Page 8
**Methods: Monitoring**			
Data monitoring	21a	Composition of data monitoring committee (DMC); summary of its role and reporting structure; statement of whether it is independent from the sponsor and competing interests; and reference to where further details about its charter can be found, if not in the protocol. Alternatively, an explanation of why a DMC is not needed	www.hislac.org
	21b	Description of any interim analyses and stopping guidelines, including who will have access to these interim results and make the final decision to terminate the trial	n/a
Harms	22	Plans for collecting, assessing, reporting, and managing solicited and spontaneously reported adverse events and other unintended effects of trial interventions or trial conduct	Page 8
Auditing	23	Frequency and procedures for auditing trial conduct, if any, and whether the process will be independent from investigators and the sponsor	Page 7
**Ethics and dissemination**			
Research ethics approval	24	Plans for seeking research ethics committee/institutional review board (REC/IRB) approval	Ethics ref 13/WA/0372
Protocol amendments	25	Plans for communicating important protocol modifications (eg, changes to eligibility criteria, outcomes, analyses) to relevant parties (eg, investigators, REC/IRBs, trial participants, trial registries, journals, regulators)	Oversight committee

For peer review only - http://bmjopen.bmj.com/site/about/guidelines.xhtml

Consent or assent	26a	Who will obtain informed consent or assent from potential trial participants or authorised surrogates, and how (see Item 32)	n/a
	26b	Additional consent provisions for collection and use of participant data and biological specimens in ancillary studies, if applicable	n/a
Confidentiality	27	How personal information about potential and enrolled participants will be collected, shared, and maintained in order to protect confidentiality before, during, and after the trial	Anonymised
Declaration of interests	28	Financial and other competing interests for principal investigators for the overall trial and each study site	None
Access to data	29	Statement of who will have access to the final trial dataset, and disclosure of contractual agreements that limit such access for investigators	n/a
Ancillary and post-trial care	30	Provisions, if any, for ancillary and post-trial care, and for compensation to those who suffer harm from trial participation	n/a
Dissemination policy	31a	Plans for investigators and sponsor to communicate trial results to participants, healthcare professionals, the public, and other relevant groups (eg, via publication, reporting in results databases, or other data sharing arrangements), including any publication restrictions	Page 11
	31b	Authorship eligibility guidelines and any intended use of professional writers	n/a
	31c	Plans, if any, for granting public access to the full protocol, participant-level dataset, and statistical code	www.hislac.org
**Appendices**			
Informed consent materials	32	Model consent form and other related documentation given to participants and authorised surrogates	n/a
Biological specimens	33	Plans for collection, laboratory evaluation, and storage of biological specimens for genetic or molecular analysis in the current trial and for future use in ancillary studies, if applicable	n/a

*It is strongly recommended that this checklist be read in conjunction with the SPIRIT 2013 Explanation & Elaboration for important clarification on the items. Amendments to the protocol should be tracked and dated. The SPIRIT checklist is copyrighted by the SPIRIT Group under the Creative Commons "Attribution-NonCommercial-NoDerivs 3.0 Unported" license.

For peer review only - http://bmjopen.bmj.com/site/about/guidelines.xhtml

6

For peer review only

A Two-Epoch Cross-Sectional Case Record Review Protocol Comparing Quality Of Care Of Hospital Emergency Admissions At Weekends Versus Weekdays

Journal:	*BMJ Open*
Manuscript ID	bmjopen-2017-018747.R1
Article Type:	Protocol
Date Submitted by the Author:	11-Oct-2017
Complete List of Authors:	Bion, Julian; University of Birmingham, Intensive Care Medicine<br>Aldridge, Cassie; University of Birmingham<br>Girling, Alan; University of Birmingham, Institute of Applied Health Research<br>Rudge, Gavin; University of Birmingham, School of Health and Population Sciences<br>Beet, Chris; University Hospitals Birmingham NHS Foundation Trust<br>Evans, Tim; NHS Improvement<br>Temple, R.; Heart of England NHS Foundation Trust, Renal Medicine<br>Roseveare, Chris; Southern Health NHS Foundation Trust<br>Clancy, Mike; University Hospital Southampton NHS Foundation Trust<br>Boyal, Amunpreet; University Hospitals Birmingham NHS Foundation Trust<br>Tarrant, Carolyn; University of Leicester<br>Sutton, Elizabeth; University of Leicester<br>Sun, Jianxia; University Hospitals Birmingham NHS Foundation Trust<br>Rees, Peter; Member of the Academy of Medical Royal Colleges Patient Liaison Group<br>Mannion, Russell; University of York, Management School<br>Chen, Yen-Fu; University of Warwick, Warwick Medical School<br>Watson, Samuel; University of Warwick, Warwick Medical School;<br>Lilford, Richard; University of Warwick, Division of Health and Population Sciences
**Primary Subject Heading**:	Health services research
Secondary Subject Heading:	Health policy
Keywords:	Hospital, weekend, quality, case record review, consultants

Title: A Two-Epoch Cross-Sectional Case Record Review Protocol Comparing Quality Of Care Of Hospital Emergency Admissions At Weekends Versus Weekdays

Authors: Bion JF*, Aldridge C, Girling A, Rudge G, Beet C, Evans T, Temple M, Roseveare P, Clancy M, Boyal A, Tarrant C, Sutton E, Sun J, Rees P, Mannion R, Chen Y-F, Watson S, Lilford R, on behalf of the HiSLAC collaboration.

1. Primary author

*Corresponding author details:
Professor Julian Bion FRCP FRCA FFICM MD
University Department of Anaesthesia & Critical Care Office 1,
Ground Floor East Heritage Building (Queen Elizabeth Hospital)
Mindelsohn Way, Edgbaston,
Birmingham B15 2TH
Tel: 0121 371 6816
Email: J.f.bion@bham.ac.uk

Writing committee:
*Professor Julian Bion[1], University of Birmingham, Birmingham, UK
Cassie Aldridge, University of Birmingham, Birmingham, UK
Alan Girling, University of Birmingham, Birmingham, UK
Gavin Rudge, University of Birmingham, Birmingham, UK
Chris Beet, University Hospitals Birmingham, Birmingham, UK
Professor Tim Evans, NHS Improvement, London, UK
Mark Temple, Heart of England NHS Foundation Trust, Birmingham, UK
Chris Roseveare, Southern Health NHS Foundation Trust, Southampton, UK
Mike Clancy, University Hospitals Southampton NHS Foundation Trust, Southampton, UK
Amunpreet Boyal, University Hospitals Birmingham, Birmingham, UK
Carolyn Tarrant, University of Leicester, Leicester, UK

For peer review only - http://bmjopen.bmj.com/site/about/guidelines.xhtml

For peer review only

Elizabeth Sutton, University of Leicester, Leicester, UK
Jianxia Sun, University Hospitals Birmingham, Birmingham, UK
Peter Rees, Member of the Academy of Medical Royal Colleges Patient Liaison Group, London, UK
Professor Russell Mannion, University of York, York, UK
Yen-Fu Chen, University of Warwick, Warwick, UK
Sam Watson, University of Warwick, Warwick, UK
Professor Richard Lilford, University of Warwick, Warwick, UK

Key Words
Hospital, weekend, quality, case record review, consultants

Word count
3858

Acknowledgement of Funding: This project was funded by the National Institute for Health Research, Health Services and Delivery Research Programme (project number 12/128/17). Theviews and opinions expressed therein are those of the authors and do not necessarily reflect those of the HS&DR, NIHR, NHS or the Department of Health.

For peer review only - http://bmjopen.bmj.com/site/about/guidelines.xhtml

ABSTRACT

Introduction: The mortality associated with weekend admission to hospital (the 'weekend effect') has for many years been attributed to deficiencies in quality of hospital care, often assumed to be due to suboptimal senior medical staffing at weekends. This protocol describes a case note review to determine whether there are differences in care quality for emergency admissions to hospital at weekends compared with weekdays, and whether the difference has reduced over time as health policies have changed to promote seven-day services.

Methods and analysis: Cross-sectional two-epoch case record review of 20 acute hospital Trusts in England. Anonymised case records of 4000 emergency admissions to hospital, 2000 at weekends and 2000 on weekdays, covering two epochs (financial years 2012-13, and 2016-17). Admissions will be randomly selected across the whole of each epoch from Trust electronic patient records. Following training, structured implicit case reviews will be conducted by consultants or senior registrars (senior residents) in acute medical specialities (60 case records per reviewer), and limited to the first seven days following hospital admission. The co-primary outcomes are the weekend:weekday admission ratio of errors per case record, and a global assessment of care quality on a Likert scale. Error rates will be analysed using mixed effects logistic regression models, and care-quality using ordinal regression methods. Secondary outcomes include error typology; error-related adverse events; and any correlation between error rates and staffing. The data will also be used to inform a parallel health economics analysis.

Ethics and dissemination: The project has received ethics approval from the South West Wales Research Ethics Committee: Reference 13/WA/0372. Informed consent is not required for accessing anonymised patient case records from which patient identifiers had been removed. The findings will be disseminated through peer-reviewed publications in high-quality journals and through local HiSLAC leads at the 121 hospitals that make up the HiSLAC Collaborative.

Methodological Strengths and Limitations

- Difference-in-difference analysis minimises bias from variations in case- mix or institutional characteristics, and allows differentiation of secular trends from the effects of the intervention (specialist intensity).

For peer review only - http://bmjopen.bmj.com/site/about/guidelines.xhtml

- Structured implicit case record review encourages a standardised approach to assessment, while permitting reviewers to exercise expert clinical judgement.
- Inter-assessor variation will be measured through duplicate review of 800 case records, and minimised though standardised training.

For peer review only

For peer review only - http://bmjopen.bmj.com/site/about/guidelines.xhtml

INTRODUCTION:

The mortality associated with weekend admission to hospital (the 'weekend effect') has for many years been attributed to deficiencies in quality of hospital care, usually linked to perceptions that senior medical staffing at weekends was suboptimal [1]. The weekend effect has been used as a justification for introducing seven-day services in England [2]. Seven-day services require hospital Trusts in England to meet particular standards for increasing the intensity of weekend senior medical staffing [3]. However, there is little evidence that deficiencies in medical staffing cause the weekend effect. Preliminary research by the High-intensity Specialist Led Acute Care (HiSLAC) collaboration [www.hislac.org] did not identify an association between weekend-weekday specialist intensity differences and weekend-weekday admission mortality rate differences across the English NHS [4]. However, this was a cross-sectional survey in which no hospital Trust achieved parity between weekend and weekday specialist staffing intensity.

Other potential causes of the weekend effect might include patient factors (severity of illness and case mix including imperfect case mix adjustment), contextual factors (resource allocation, health policy, secular trends), and care processes (quality of care). Several studies have identified greater severity of illness amongst weekend admissions [5,6,7,8,9], while another reports increased mortality associated with weekend discharge [10]: these suggest a potential community contribution to the weekend effect. Evidence that quality of care in hospital might be worse at weekends comes from an analysis of an Australian regional voluntary critical incident reporting system [11], but this did not take into account the potential for severity of illness to enhance the opportunity for error, and did not characterize the nature of the critical incidents.

If suboptimal consultant ('specialist') staffing were indeed a cause for the weekend effect, as implied by the 7-day standards policy initiative, there are several ways this might be revealed. First, health care error rates might be higher amongst patients admitted at weekends; second, error typology would include those most likely to be mitigated by the presence of senior physicians (diagnostic accuracy, treatment specificity and timeliness of care); third, a positive association might be expected between the difference in weekend:weekday error rates and the difference in weekend:weekday specialist intensities, indicating that at weekends less reliable care is associated with reduced senior physician presence; and fourth, that weekend performance and staffing indices improve with the introduction of 7-day services focused on maximizing specialist staffing.

We describe here a protocol for comparing quality of care given to patients admitted as emergencies to 20 hospital Trusts at weekends and on weekdays during two epochs, representing periods before (2012-2013) and during (2016-17) the implementation of NHS England's seven-day services standards. The

For peer review only - http://bmjopen.bmj.com/site/about/guidelines.xhtml

outputs from this protocol will be integrated with parallel HiSLAC research workstreams in specialist intensity [4], a systematic review and framework synthesis [12], ethnography [13], and health economics [14].

Aims and objectives

Using retrospective review of case records from 20 hospital Trusts during two time epochs, this study aims to determine whether there is a difference in quality of care offered to patients undergoing emergency admission to hospital at weekends compared with weekdays.

The main objectives are:

1. to compare rates of errors and differences in care quality between weekend and weekday admissions
2. to examine prevalent error types for weekend and weekday admissions
3. if a difference in error rates or care-quality between weekend and weekday admissions is found, to examine whether the difference has changed between the two epochs
4. to inform the Bayesian model proposed in our parallel health economics model [14]

The co-primary outcomes are the weekend-weekday error rate ratio and the global assessment of care quality. The error rate is calculated as the number of errors per case record from admission to discharge or to seven days, whichever occurs first.

Secondary outcomes include a comparison of weekend-weekday admissions in error typology and error-related adverse events; of error rates by day of the week within each admission group; and correlation of weekend:weekday error rate ratios with weekend:weekday differences in specialist hours per 10 emergency admissions (EAs).

METHODS AND ANALYSIS

We will conduct a cross-sectional two-epoch comparison of care quality received by emergency patients admitted to hospital at weekends and on weekdays, employing a difference-in-difference analysis [15] to minimise bias from between-Trust differences in case mix and staffing. Conceptually,

For peer review only - http://bmjopen.bmj.com/site/about/guidelines.xhtml

seven-day services is the research domain, and 'HiSLAC implementation' the research intervention, manifest as an increase in specialist staffing at weekends.

Participating hospital Trusts:

From the 121 acute hospital Trusts participating in HiSLAC, 20 will be invited to participate based on Trust size and Sunday specialist intensity derived from the HiSLAC annual point prevalence survey [4]. Trusts will first be grouped into size quintiles based on the number of beds. Within each quintile, hospitals will be ranked by Sunday specialist intensity (hours per 10 emergency admissions) and two Trusts selected from the top and bottom of the intensity spectrum. In the largest Trust quintile, we will also match on number of acute hospitals within each Trust to ensure balance between single-site and multiple-site Trusts.

Reviewer Recruitment and training

We will contact all participating Trusts in England to invite a total of 80 consultants or registrars (residents) in year 5 or above of their training, in an acute adult medical speciality, to act as case record reviewers. Registrar reviewers will conduct their reviews under the indirect supervision of each Trust's HiSLAC local project lead (LPL, a senior consultant (attending)). The reviewers will undertake a training course covering the principles of case reviews, providing familiarisation with the scanned case records, and permitting practice with samples of case records allowing feedback and discussion.

Patient case record selection*:*

We will evaluate 4000 non-operative emergency admissions to 20 Trusts to measure variations in quality of care between weekends and weekdays, between high-intensity and low-intensity Trusts, and over time. Case records will be randomly selected from across the whole period of two epochs: financial year 1st April 2012-31st March 2013, and 1st April 2016-31st March 2017, to capture the pre-policy and post-policy intervention periods of specialist staffing respectively, and to avoid bias from seasonal variation. Each epoch will include 100 case records of adult (age 17 years or more) emergency non-operative admissions from each of 20 hospitals, 10 with high and 10 with low-intensity specialist led care (2000 case records for each epoch, 4000 case records in total). Half the records from each centre will be weekend admissions (midnight Friday to midnight Sunday), half weekday. We have chosen non-

For peer review only - http://bmjopen.bmj.com/site/about/guidelines.xhtml

operative admissions because the surgical population is currently the subject of a separate national multifaceted quality improvement programme, EPOCH (http://www.epochtrial.org/). The analysis is not restricted to mortality reviews, as it is important to avoid endogenous selection bias (from the outcome influencing the sample). Moreover, deaths are not representative of the general in-patient population [16], and avoidable deaths formed only 3.6% of 3400 records in a recent review [17] (less than 1% when adjusting for inconsistency between reviewers [18]).

Case Record Processing (Fig 1):

Patient records will be selected randomly at the University of Birmingham (UoB) from Patient Administration System (PAS) patient records which will have been anonymised at each collaborating Trust, allocated an encrypted code, and transmitted by secure file transfer protocol to be stored on a secure server at UoB. As case records may be missing, Trusts will be provided with 260 codes of which 65 will be weekend and 65 weekday admissions for each epoch; record retrieval will stop once 50 have been obtained in each group (200 total). Following case selection, the encrypted code will be sent to the Trust medical records department, converted to the local NHS number, and the records retrieved for masking to remove patient identifiers.

Only the first seven days following admission will be copied, as the purpose of the review is to focus on care related to the admission phase, not the totality of the patient's course in hospital. A window of seven days will capture most of the medical care targeted at the acute disease process precipitating admission, and associated adverse events linked to the admission. The redacted records will then be scanned as pdf documents, and transmitted by secure file transfer protocol to UoB (Fig 1). Thereby, case records will be anonymised and digitised before being presented to the reviewers.

The Review Process (Fig 2 and Appendix):

We will employ retrospective structured implicit case record review (SICRR) to detect errors in care [17, 19, 20], inviting reviewers to produce short narrative judgements with a 5-point Likert scale rating of overall care quality. Reviewers will be asked to identify the location and date of any errors, associate them with specific domains of care (e.g: investigation, diagnosis, monitoring, etc), and to make a judgement about whether the errors caused harm to the patient in terms of adverse events.

Four thousand case records will be reviewed (100 from each epoch, 200 total from each of 20 trusts). Each Trust's collection of 200 case records will be divided into three (non-overlapping) sets of 80 records as follows: Set 1 is a random collection of 80 case records from Epoch 1; Set II is a random collection

For peer review only - http://bmjopen.bmj.com/site/about/guidelines.xhtml

of 80 case records from Epoch 2; and Set III is the remaining 40 case records (20 from each epoch), with each case record duplicated to create 80 records. Each of the 80 reviewers will be randomly allocated just one case record from each of the three sets above, that is, three case records from each Trust. Under this scheme each reviewer performs 60 reviews and each Trust contributes 40 case records to be reviewed twice; inter-rater reliability can be assessed from the 800 (20%) case-notes with independent reviews from different reviewers using the global ratings of care quality.

Data extraction:

Reviewers will access case records on-line. The case notes will be presented in random order so that learning and fatigue cannot bias the results. Reviewers will not be informed whether records relate to weekend or weekday admissions, but dates will not be masked from the copied records because of the need to determine the timing of events. Reviewers will work independently; they may discuss interpretation of case records with the senior clinician when there is uncertainty, but will be asked to refrain from discussing case records with each other: discussion between reviewers does not improve reliability [21]. The large number of reviewers contributing to each epoch will improve 'calibration' i.e.; reduce the effect of any outliers ('hawks' and 'doves'). After completion of 10 reviews we will compare the number of errors identified and global ratings from each of the 80 reviewers to see if there are any extreme hawks or doves.

Data extraction will be performed by the reviewers using a standardised web-based reporting template (Appendix 2, based on prior work by Hogan [17] and others [22, 23], and on the national Mortality Review process [20]). Assessment of quality of care will be censored at seven days following admission, because the primary aim of the analysis is to examine factors around the time of admission, not later in the patient's stay. Reviewers will be asked to note the date and time (from which elapsed time from admission can be calculated) of any identified errors, so that proximity to the admission point can be recorded. Reviewers will note only those adverse events associated with errors, and assess preventability.

Process and Outcome Metrics

Errors of omission or commission will be defined as '*the failure of a planned action to be completed as intended or use of a wrong, inappropriate, or incorrect plan to achieve an aim*' (World Health Organisation's taxonomy [24]). Reviewers will be asked to categorise the errors they identify, permitting subsequent comparisons of typology of error (Appendix 1) in relation to context (weekends, weekdays, night versus day).

For peer review only - http://bmjopen.bmj.com/site/about/guidelines.xhtml

Adverse events are defined as ‘*An event that results in unintended harm to the patient by an act of commission or omission rather than by the underlying disease or condition of the patient’* [24, 25]. Reviewers will grade the preventability of the adverse event using a standard 6-point scale [26]. This approach permits assessment of the consequences of the errors, without asking reviewers to collect information on all adverse events, many of which will have arisen as a recognised complication or consequence of illness rather than from a lapse in clinical care.

Global (implicit) measure of quality: reviewers will provide an overall assessment of care quality, using a 5-point Likert scale in response to the question ‘To what extent did this patient receive best practice care?”

Specialist intensity (‘HiSLAC Implementation’): Specialist (consultant) direct involvement in patient care will be derived from the annual High-intensity Specialist Led Acute Care (HiSLAC) point prevalence survey [4], and expressed as specialist hours per 10 emergency admissions. This self-report web-based survey is administered in June each year; all specialists in 121 participating Trusts across England are invited to record whether they were in the hospital on a particular Sunday or the following Wednesday providing direct clinical care to patients admitted as emergencies, and if so, how many hours they spent doing so.

ANALYTICAL PLAN:

Quality of care:

Error: The primary metric is the number of errors per case record (ie: per episode of care) from admission to discharge or to seven days, whichever occurs first. The weekend-weekday admission error rate ratio will determine whether weekend admission is associated with more errors in care than weekday admission. We will also examine whether weekend-weekday error rate differences and global assessments of care quality vary by admission epoch and degree of HiSLAC implementation (specialist hours per 10 emergency admissions). We will look for a difference in the difference between weekdays and weekends across low and high intensity hospitals, and a further difference between epochs. In this way each hospital acts as its own control, thereby adjusting for variation in institutional case mix. We hypothesize that Epoch 1 error rates (the proportion of case records with one or more errors) will be in the region of 20%, and that this rate will diminish between epochs. We will also perform secondary analyses to determine whether errors are more frequent at weekends than weekdays.

For peer review only - http://bmjopen.bmj.com/site/about/guidelines.xhtml

Adverse event (AE) rates will be analysed similarly. AEs have been reported consistently to affect around 10% of patient episodes, of which half are reported as being preventable [26]. As we will only document adverse events considered to be associated with an error in care, the incidence of adverse events in our data set is likely to be lower than 10%, but the preventability rate higher.

Error Typology: Error types will be classified by the reviewers using the coding in Appendix 1. In addition we will perform a qualitative comparison of the difference between reviewers in their typology of errors. Reviewers' free text descriptions of error events will be imported into NVivo for further comparative analysis based on a coding frame developed from a sample of transcripts reviewed and discussed by the members of the project team (including clinical and non-clinical members). The focused coding frame will then be applied to the free-text descriptors to derive a separate classification of error types, permitting a comparison between the a priori classification (derived from Hogan et al [17]) and a grounded approach.

Health economics: We will use these data to inform the development of the Bayesian model proposed in our parallel health economics modelling [14]. The parameter estimates obtained for error rates from the difference in difference in difference type of analysis will be used to update a prior probability distribution obtained from experts. The elicitation of these prior probabilities will be informed by data from the HiSLAC mixed-methods systematic review of the weekend effect [12] and information derived from the parallel HiSLAC ethnographic study of the 20 hospitals [13]. The methodology for elicitation of this 'prior' and for the updating process has been described elsewhere [14]. In order to calculate the cost utility/benefit of the 7-day services policy of increasing specialist intensity, three further parameters are needed: a) the error rates must be converted into adverse event rates and hence into QALYs using the method of Yao et al [27] and Lilford et al [28]; b) potential cost savings from (any) reduction in adverse events will be calculated, again according to Yao et al [27]; and c) the cost of the intervention itself will be calculated building on Meacock et al [29]. Net costs will be derived by subtracting cost (b) from cost (c). The intervention will dominate if these are negative (since such a scenario can only arise if there is a substantial decrease in adverse events).

Statistical Analysis

Rates of errors and adverse events will be analysed using mixed effects logistic regression models; and the quality of care Likert scale analysed using mixed effects ordinal logistic models, supplemented by linear analyses of the ordinal values. The models will incorporate additive random effects for case-notes, reviewers and hospital trusts and fixed effects for day of week (weekend/weekday), time epoch and their interaction. Trust-level weekend effects will be extracted from these analyses. Changes in trust-level effects between epochs will be correlated with contemporaneous changes in specialist involvement estimated from the point prevalence survey.

For peer review only - http://bmjopen.bmj.com/site/about/guidelines.xhtml

Reviewer reliability coefficients will be computed as the proportion of the total variance due to variation between case-notes.

Power and detectable differences

The balanced nature of the design ensures that reviewer and Trust effects are eliminated from the comparisons of interest, at least to a good approximation. Based on earlier work [18] we may assume that 30% of the remaining variance will be attributable to variation between case-notes. From Hutchison et al [19], the base error rate is taken as 20% and the standard deviation of the Likert global quality scale estimated to be 1.06 (this value is adjusted to take account of the smaller number of Likert categories used in the current study – i.e. 5 instead of 6).

Using a P-value of 5%, the following effects will be detectable with 80% power: (a) a difference in weekend/weekday error rates of 3.2% (from 20 trusts, both epochs); (b) a change over time in the weekend/weekday difference of 6.4%; (c) an average difference between weekend and weekday Likert care scores of 0.08 (which may be interpreted as a shift of one Likert category for 1 case in every 12; and (d) a change over time in this difference of 0.17.

DISCUSSION

This protocol describes using retrospective case record review to determine whether the quality of care received by patients admitted to hospital as emergencies, varies between weekdays and weekends. The study will contribute to our understanding of the 'weekend effect' and the implementation of seven-day services policy by integrating data on error rates and adverse events with parallel workstreams on specialist staffing [4], a systematic review and framework synthesis of the weekend effect [12], ethnographic evaluations [13], and Bayesian health economics modelling [14].

The strengths of this novel approach lie in triangulating multiple sources of information on the implementation of national health policy, and the use of difference-in-difference analyses to minimise bias from variations in institutional structures and case mix.

Case record review is an imperfect instrument for measuring quality of care. Conventional approaches include explicit criterion-based methods (framed by a checklist) or implicit judgement-based methods. While both are widely used, there are important differences between them. Criterion-based review generally provides a higher level of inter-rater agreement than implicit review, but has been criticised for its insensitivity to detecting more nuanced

For peer review only - http://bmjopen.bmj.com/site/about/guidelines.xhtml

aspects of care quality, and because the criteria themselves may be susceptible to selection bias. The implicit approach allows reviewers to exercise expert clinical judgement in identifying determinants of quality which may have been omitted from pre-defined criteria, such as evaluating accuracy of diagnosis and appropriateness of care pathways, but this requires a certain level of clinical experience. Midway between these two approaches is the structured implicit case record review (SICRR) [17, 19, 20], which is the method we will adopt here. Recommended for the national mortality review process supported by the Royal College of Physicians [20], SICRR invites reviewers to produce short narrative judgements of care quality in specific phases of care, with a 6-point rating of care quality. The case record review process follows that adopted by Benning et al [30], using explicit (criterion based) and implicit (holistic) approaches since they identify a different spectrum of errors [31, 32]. Implicit review is essential to this study since specialist care is most likely to impact on selecting the correct clinical pathway through accurate diagnosis rather than adhering to that pathway once identified, which is where explicit review has its focus.

Our primary measure of quality will be error rates. We have chosen to focus on processes of care rather than outcomes (adverse events) because process assessment provides a broader foundation on which to assess safety and quality of care, whereas adverse events may arise from the natural history of the disease [16, 31, 32, 33]. We recognise that reliability between reviewers may be higher for diseases with a strong evidence base [34] and weaker for the likely diverse set of conditions associated with emergency admission to hospital as in this review. To mitigate this effect reviewers will be asked to categorise the errors they identify, permitting subsequent comparisons of typology of error (Appendix 1).

Reviewers will also be asked to document whether or not the errors they identify were associated with an adverse event, thereby linking process to outcome [35], and to grade the preventability of the adverse event using a standard 6-point scale [27]. This permits assessment of the consequences of the errors, without asking reviewers to collect information on all adverse events regardless of causation.

We will not restrict the analysis to mortality reviews, because although adverse events (AEs) and preventable AEs are twice as frequent amongst hospital non-survivors as survivors, and error rates might therefore also be over-represented in this population, these are not representative of the general in-patient population [16]. Random selection of case records from all non-operative admissions will minimise risk of bias which might be associated with random selection of deaths, as the intervention may change the risk of the outcome which would also change the chance of being in the sample (endogenous selection).

ETHICS AND DISSEMINATION

For peer review only - http://bmjopen.bmj.com/site/about/guidelines.xhtml

The HiSLAC study, including the case record reviews, was approved by the Review Sub-committee of the South West Wales REC on 11 November 2013. Informed consent was not required for accessing anonymised patient case records from which patient identifiers had been removed. The findings of this study will be published in peer-reviewed journals, the outputs from this research will also form part of the project report to the HS&DR Programme Board.

Competing interests

JB is the Chair, NICE Acute Medical Emergencies Guideline Development Group
National Clinical Guideline Centre. All other authors declare no competing interests.

Data sharing statement

We shall make findings available to the scientific community with as few restrictions as feasible when the study has been completed.

Author Contributions

JB and RL developed the initial study idea. CA, JB and AB obtained statutory and ethics approval. JB, CA, AG, GR, CB, TE, MT, CR, MC, AB, CT, ES, JS, PR, RM, YC, SW and RL informed the protocol methodology. All authors contributed to writing the final report and approved the final version.

For peer review only - http://bmjopen.bmj.com/site/about/guidelines.xhtml

REFERENCES

1. Bell CM, Redelmeier DA. Mortality among patients admitted to hospitals on weekends as compared with weekdays. N Engl J Med. 2001 Aug 30;345(9):663-8.
2. Hansard, 13th Oct 2015. https://www.publications.parliament.uk/pa/cm201516/cmhansrd/cm151013/debtext/151013-0001.htm
3. NHS England 2013. https://www.england.nhs.uk/ourwork/qual-clin-lead/seven-day-hospital-services/the-clinical-case/
4. Aldridge C et al. Weekend specialist intensity and admission mortality in acute hospital trusts in England: a cross-sectional study. The Lancet 388.10040 (2016): 178-186.
5. Meacock R, Anselmi L, Kristensen SR, Doran T, Sutton M. Higher mortality rates amongst emergency patients admitted to hospital at weekends reflect a lower probability of admission. J Health Serv Res Policy 2016; May 6. pii: 1355819616649630.
6. Mikulich O, Callaly E, Bennett K, O'Riordan D, Silke B. The increased mortality associated with a weekend emergency admission is due to increased illness severity and altered case-mix. Acute Med. 2011;10(4):182-7.
7. Vest-Hansen B, Riis AH, Sørensen HT, Christiansen CF. Out-of-hours and weekend admissions to Danish medical departments: admission rates and 30-day mortality for 20 common medical conditions. BMJ Open 2015 Mar 11;5(3):e006731.
8. Goodman EK, Reilly AF, Fisher BT, Fitzgerald J, Li Y, Seif AE, Huang YS, Bagatell R, Aplenc R. Association of weekend admission with hospital length of stay, time to chemotherapy, and risk for respiratory failure in pediatric patients with newly diagnosed leukemia at freestanding US children's hospitals. JAMA Pediatr 2014;168(10):925-31.
9. Anselmi L, Meacock R, Kristensen SR, Doran T, Sutton M. Arrival by ambulance explains variation in mortality by time of admission: retrospective study of admissions to hospital following emergency department attendance in England. BMJ Qual Saf Published Online First: 18 October 2016. doi: 10.1136/bmjqs-2016-005680
10. Sayers A, Whitehouse MR, Berstock JR, Harding KA, Kelly MB, Chesser TJ. The association between the day of the week of milestones in the care pathway of patients with hip fracture and 30-day mortality: findings from a prospective national registry – The National Hip Fracture Database of England and Wales. BMC Medicine 2017; 15:62.
11. Buckley D, Bulger D. Trends and Weekly and Seasonal Cycles in the Rate of Errors in the Clinical Management of Hospitalized Patients. Chronobiology International 2012; 29:7, 947-954

For peer review only - http://bmjopen.bmj.com/site/about/guidelines.xhtml

12. Chen YF, Boyal A, Sutton E, Armoiry X, Watson S, Bion J, Tarrant C; HiSLAC Collaborative. The magnitude and mechanisms of the weekend effect in hospital admissions: A protocol for a mixed methods review incorporating a systematic review and framework synthesis. Syst Rev. 2016 May 21;5(1):84.
13. Tarrant C, Sutton E, Angell E, Aldridge CP, Boyal A, Bion J on behalf of the HiSLAC study. The 'weekend effect' in acute medicine: a protocol for a team-based ethnography of weekend care for medical patients in acute hospital settings. BMJ Open 2017;7:e016755. doi: 10.1136/bmjopen-2017-016755
14. Watson S et al – Protocol for the health economic evaluation of increasing the weekend specialist to patient ratio submitted 2017 BMJ Open.
15. Sutton M, Nikolova S, Boaden R, Lester H, McDonald R, Roland M. Reduced mortality with hospital pay for performance in England. N Engl J Med. 2012 Nov 8;367(19):1821-8.
16. Baines RJ, Langelaan M, de Bruijne MC, Wagner C. Is researching adverse events in hospital deaths a good way to describe patient safety in hospitals: a retrospective patient record review study. BMJ Open 2015 Jul 9;5(7):e007380. doi: 10.1136/bmjopen-2014-007380.
17. Hogan H, Zipfel R, Neuburger J, Hutchings A, Darzi A, Black N. Avoidability of hospital deaths and association with hospital-wide mortality ratios: retrospective case record review and regression analysis. BMJ. 2015 Jul 14;351:h3239. doi: 10.1136/bmj.h3239.
18. Manaseki-Holland S, Lilford RJ, Bishop JRB, et al. Reviewing deaths in British and US hospitals: a study of two scales for assessing preventability. BMJ Qual Saf Published Online First: 22 June 2016. doi: 10.1136/bmjqs-2015-004849
19. Hutchinson A, Coster JE, Cooper KL, Pearson M, McIntosh A, Bath PA. A structured judgement method to enhance mortality case note review: development and evaluation. BMJ Qual Saf. 2013 Dec;22(12):1032-40. doi: 10.1136/bmjqs-2013-001839.
20. Royal College of Physicians. Using the structured judgment review method – a clinical governance guide to mortality case record reviews. London: RCP, 2016.
21. Hofer TP, Bernstein SJ, DeMonner S, Hayward RA. Discussion between Reviewers Does Not Improve Reliability of Peer Review of Hospital Quality. Medical Care 2000; 38(2): 152-161
22. Vincent, C., G. Neale, and M. Woloshynowych, Adverse events in British hospitals: preliminary retrospective record review. BMJ, 2001. 322: p. 517-9.
23. Hayward, R. and T. Hofer, Estimating Hospital Deaths Due to Medical Error- Preventability is in the eye of the reviewer. JAMA, 2001. 286(4): p. 415-20.
24. WHO 2009. Final Technical Report for The Conceptual Framework for the International Classification for Patient Safety v.11. Technical Annex 2 - Glossary of Patient Safety Concepts and References. www.who.int/patientsafety/taxonomy/icps_technical_annex2.pdf
25. Aspden P, Corrigan J, Wolcott J, Erickson S, editors. Institute of Medicine, Committee on Data Standards for Patient Safety, Board on Health Care Services. Patient Safety: Achieving a New Standard for Care. Washington DC: National Academies of Sciences, 2004.

For peer review only - http://bmjopen.bmj.com/site/about/guidelines.xhtml

26. Mayor S, Baines E, Vincent C, Lankshear A, Edwards A, Aylward M, et al. Measuring harm and informing quality improvement in the Welsh NHS: the longitudinal Welsh national adverse events study. Health Serv Deliv Res 2017;5(9)
27. Yao GL, Novielli N, Manaseki-Holland S, Chen Y-F, van der Klink M, Barach P, Chilton PJ, Lilford RJ. Evaluation of a predevelopment service delivery intervention: an application to improve clinical handovers. BMJ Qual Saf 2012; 21 Suppl 1: i29-38.
28. Lilford RJ, Girling AJ, Sheikh A, Coleman JJ, Chilton PJ, Burn SL, Jenkinson DJ, Blake L, Hemming K. Protocol for evaluation of the cost-effectiveness of ePrescribing systems and candidate prototype for other related health information technologies. BMC Health Serv Res 2014; 14(1): 314.
29. Meacock R, Doran T, Sutton M. What are the Costs and Benefits of Providing Comprehensive Seven-day Services for Emergency Hospital Admissions? Health Econ. 2015 Aug;24(8):907-12. Doi: 10.1002/hec.3207. Epub 2015 May 22.
30. Benning A, Ghaleb M, Suokas A, Dixon-Woods M, Dawson J, Barber N, Franklin BD, Girling A, Hemming K, Carmalt M, Rudge G, Naicker T, Nwulu U, Choudhury S, Lilford R. Large scale organisational intervention to improve patient safety in four UK hospitals: mixed method evaluation. BMJ. 2011 Feb 3;342:d195. doi: 10.1136/bmj.d195.
31. Lilford RJ, Brown CA, Nicholl J. Use of process measures to monitor the quality of clinical practice. BMJ. 2007 Sep 29;335(7621):648-50.
32. Lilford RJ, Mohammed MA, Braunholtz D, Hofer TP. The measurement of active errors: methodological issues. Qual Saf Health Care. 2003 Dec;12 Suppl 2:ii8-12.
33. Hofer TP. Commentary on Sharek: adverse events and errors-important to differentiate and difficult to measure. Health Serv Res. 2011 Apr;46(2):679-84.
34. Hofer TP, Asch SM, Hayward RA, Rubenstein LV, Hogan MM, Adams J, Kerr EA. Profiling quality of care: Is there a role for peer review? BMC Health Serv Res. 2004 May 19;4(1):9.
35. National Patient Safety Agency. Seven Steps to Patient Safety - your guide to safer patient care. NPSA London 2004

For peer review only

For peer review only - http://bmjopen.bmj.com/site/about/guidelines.xhtml

FIGURES

Fig 1: Case record processing

Fig 2: The Review Process

APPENDICES

APPENDIX 1: Reference Material
APPENDIX 2: HiSLAC Case Record Review Data Collection

For peer review only

For peer review only - http://bmjopen.bmj.com/site/about/guidelines.xhtml

Figures

Fig 1: Case record processing

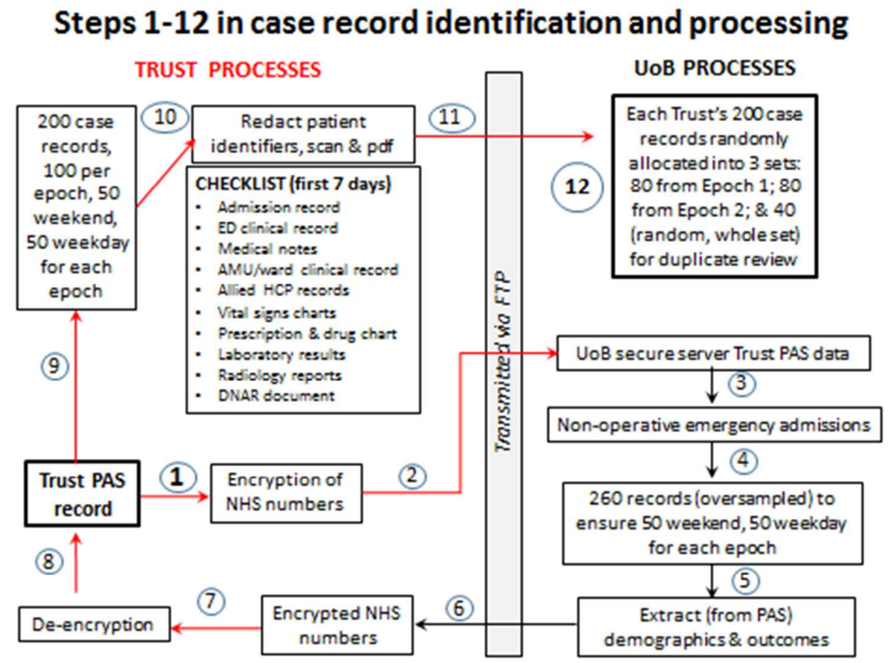

Fig 2: The Review Process

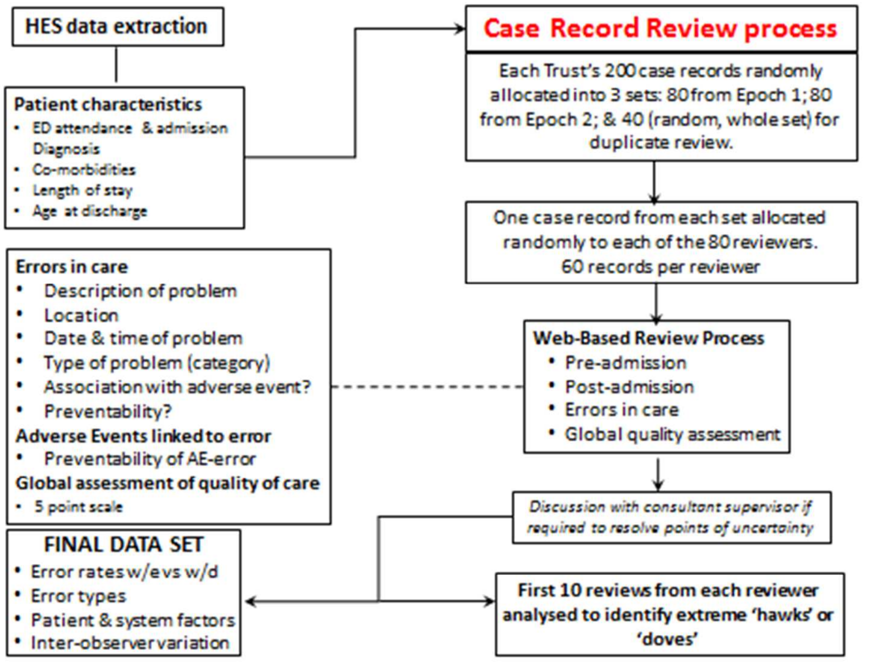

APPENDICES

APPENDIX 1: REFERENCE MATERIALS

**1.1 Location Of Error: Where did the error occur?**
A. Outside hospital (primary care, ambulance, etc.)
B. In the ED or linked area before admission
C. AMU or equivalent area
D. Acute ward (other than AMU)
E. Speciality ward (ICU/HDU, coronary care, renal, respiratory, elderly care, rehab)
F. Diagnostic area, radiology

**1.2 Error Typology**	***Examples only (not exhaustive – not for recording)***
A. Assessment, investigation or diagnosis	Physical examination and history taking incomplete<br>Pressure ulcer risk not assessed/incorrectly assessed<br>VTE risk assessment not completed/incorrectly completed<br>Falls history/vulnerability to falls not identified<br>Swallowing safety not assessed/incorrectly assessed<br>Tests and investigations missed/delayed/wrong<br>Diagnosis missed/delayed/wrong<br>Failure to assess comorbidities or frailty
B. Medication	Over- or under-hydration<br>Oxygen supply wrong/delayed/omitted<br>Allergic/anaphylactic reaction to any medication<br>Anticoagulants/antiplatelets wrong/delayed/omitted<br>Antibiotics wrong/delayed/omitted<br>Insulin or other diabetes medication wrong/delayed/omitted<br>Opiates wrong/delayed/omitted<br>Sedatives/hypnotics/antipsychotics wrong/delayed/omitted<br>Steroids wrong/delayed/omitted<br>NSAID wrong/delayed/omitted<br>Diuretics wrong/delayed/omitted<br>Antihypertensives wrong/delayed/omitted<br>Cardiovascular medications wrong/delayed/omitted<br>Chemotherapy wrong/delayed/omitted

For peer review only

C. Treatment and management plan	Appropriate medical/surgical treatment not planned<br>Avoidable delay/omission of planned medical/surgical treatment<br>Inappropriate/unnecessary medical/surgical treatment given<br>Inappropriate ceiling of care<br>Omitted/delayed/wrong treatment from AHPs<br>Acquired pressure ulcer: prevention below acceptable standard<br>Acquired pressure ulcer despite apparently acceptable standard of prevention<br>Slip/trip/fall: prevention plan below acceptable standard<br>Slip/trip/fall despite apparently acceptable standard of falls prevention<br>Developed VTE: prophylaxis below acceptable standard<br>Developed VTE despite apparently acceptable standard of VTE prophylaxis
D. Infection control	Surgical wound infection<br>Infection from invasive procedure other than surgery<br>Other healthcare associated wound infection *(e.g. infected ulcer)*<br>Infection from indwelling device *(catheter, central lines, etc.)*<br>Healthcare associated clostridium difficile<br>Healthcare- /device-associated MRSA bloodstream infection<br>Other bloodstream infection (not MRSA)<br>Healthcare associated pneumonia/chest infection (including aspiration)<br>Healthcare associated norovirus/D&V
E. Invasive procedures	Avoidable delay in undertaking procedure<br>Inadequate pre-procedure assessment/preparation<br>Complication of anaesthesia/sedation including airway management<br>Complication of operative procedure *(e.g. perforation, haemorrhage)*<br>Complication of invasive procedure *(e.g. perforation, haemorrhage)*
F. Monitoring	Vital signs monitoring.<br>Fluid intake/output.<br>Nutritional intake.<br>Delay in initiating resuscitation

For peer review only

G. Cardiopulmonary resuscitation	Delay in initiating cardiopulmonary resuscitation<br>Inappropriate resuscitation<br>Airway management<br>Resuscitation equipment
H. Other problem	Describe in free text

**1.3: Grading Of Preventability Of Adverse Event:**
1. Virtually no evidence for preventability.
2. Slight to modest evidence of preventability.
3. Possibly preventable, but not very likely (less than 50–50, but close call).
4. Probably preventable (more than 50–50, but close call).
5. Strong evidence for preventability
6. Virtually certain evidence of preventability.

**1.4: Global assessment of quality of care**
To what extent did this patient receive best practice care? (select one only)
• Completely
• Substantially
• Partially
• Very little
• Not at all

For peer review only

Appendix 2: HiSLAC Case Record Review Data Collection

On registration of each reviewer, we will allocate an unique Reviewer code which will function as log-in user name and link to the details below:

Name:	
Email:	
Phone number:	
Date of Birth (age)	
Gender	
Grade: Specialist Registrar training years 5-7, or Consultant	

Year of graduation:
Current speciality/specialities (CCT programmes) –drop-down list

TO BE RECORDED AUTOMATICALLY AT THE BEGINNING OF EACH CASE RECORD REVIEW

- Patient unique study number:
- Reviewer's Code
- Date and time of the review:

DATA COLLECTION

1. HES DATA EXTRACTED BY PROJECT TEAM BEFORE CASE RECORD REVIEW (not made available to reviewers)	
Sex M/F	
Age at hospital admission	
Date and Time of patient's first arrival at hospital (ED or other primary receiving ward).	
Duration of stay in ED (interval between arrival at hospital and time of admission, hrs & mins)	
Time, date and day of admission	
Length of hospital stay (days)	
Primary admitting diagnosis	
Comorbid disease (Charlson)	
Hospital Outcome: death or survival	

2. CLINICAL DATA EXTRACTED BY CASE RECORD REVIEWER (independently for duplicate reviews)	
1. PRE-ADMISSION PHASE INCLUDING EMERGENCY DEPARTMENT	
Source of admission:<br>• Own home<br>• Nursing or residential care home<br>• Another hospital<br>• No fixed abode<br>• No information available	

For peer review only

Patient condition immediately before the illness that led to this admission.<br>• Independent<br>• Needing help with some activities of daily living (ADLs)<br>• Dependant on others for most/all ADLs including personal hygiene<br>• Unable to determine; no relevant information in notes	
Referral mechanism:<br>• Self-presentation to ED (walk-in/own transport)<br>• 999/ambulance transfer to ED<br>• GP or deputising service referral<br>• Unable to determine	
Admission pathway:<br>Was the patient initially assessed in ED or any other short term emergency pre-admission assessment unit (e.g. Clinical Decision Unit, Ambulatory care, Medical or Surgical Assessment Unit, etc.), or was the patient admitted directly to an acute ward (AMU, general or specialty ward)?<br>• ED/pre-admission area<br>• Direct admission to acute ward<br>• Unable to determine	
2. **POST-ADMISSION PHASE**	
Location immediately following admission:<br>• Clinical Decision Unit or short stay ward<br>• Acute Medical Unit (AMU/MAU)<br>• General medical ward<br>• Medical sub-specialities including high care (eg: Coronary Care Unit, Renal Unit, Respiratory, Haematology, Oncology)<br>• Older People's Medicine/Elderly Care Unit<br>• Rehabilitation<br>• Critical Care Unit / Intensive Care Unit (including High Dependency)<br>• General Surgery (including surgical assessment/operating theatre)<br>• Other (please specify):<br>• Unable to determine ward type	
Was this an appropriate type of ward for the patient's condition?<br>• Yes, definitely appropriate<br>• Probably appropriate<br>• No<br>• Unable to determine	
Were vital signs recorded for calculation of a National Early Warning Score in the first 12 hours following admission?<br>• Yes, full vital signs and a NEWS recorded<br>• Full vital signs, but NEWS not documented<br>• Some vital signs not documented, no NEWS<br>• No evidence of vital signs or NEWS	
Initial Consultant Review in the first 24 hours following admission:	

For peer review only

- Consultant review documented [REVIEWER TO RECORD TIME AND DATE]
- Probable consultant review but status of doctor uncertain [REVIEWER TO RECORD TIME AND DATE]
- Consultant review, time not documented, but case record suggests < 14 hrs after admission
- Consultant review, time not documented, but case record suggests > 14 hrs after admission
- Unlikely that consultant review occurred during first 24 hours
- No evidence for consultant review in first 24 hours

Palliative and end-of-life care (within first 7 days): were discussions held or decisions made to limit treatment, forego resuscitation (DNACPR), or refer to palliative care?

- No: not required, patient appropriately for full treatment
- No, but would probably have been appropriate to consider some form of treatment limitation
- No, but would definitely have been appropriate to limit treatment.
- Yes: time and date of discussion or decision to limit treatment
    - Yes, appropriate decision
    - Yes, but patient might have benefited from escalation
    - Yes, but likely inappropriate decision, patient should have been considered for full escalation
- If referral to palliative care, time and date referral made:

Admission avoidance: Could admission to hospital have been avoided given optimal care in the community (eg: primary care, Hospital at Home, palliative care etc)?

- Yes
- Possibly
- No

Free Text Comments: Anything else you want to mention about this case?

3. ASSESSMENT OF CARE QUALITY

Errors in care:

Does the case record contain evidence of one or more errors in care defined as '*the failure of a planned action to be completed as intended or use of a wrong, inappropriate, or incorrect plan to achieve an aim*' (This may include events identified above). If Yes, please complete next table, errors in healthcare:

- Description of event
- Where did the event occur?
- When did the event occur?
- Category of event

For peer review only

• Associated with adverse event?<br>• If yes, grade preventability	
Proceed to next table, errors in care	

For peer review only

Errors in care. Please complete the following table using the categories listed below this table

- **An error in care is defined as** ‘the failure of a planned action to be completed as intended or use of a wrong, inappropriate, or incorrect plan to achieve an aim’**.** Errors may or may not have direct consequences for the patient, but they should have the potential to have adverse consequences. To identify errors**,** consider what an acceptable standard of healthcare would be for this patient, and describe whether (and how) the healthcare they received fell below this acceptable standard (whether through commission, omission, delay or incorrect actions). **Only one error should be entered per row.**
- **Where did the error occur (Appendix 1.1)?**
- **When did the error occur (enter date and time)**
- **What was the category of error (Appendix 1.2)? More than one may be chosen, in which case please list the most important first.**
- **Was the error associated with an adverse event (AE)? We define an AE as** ‘*An event that results in unintended harm to the patient by an act of commission or omission rather than by the underlying disease or condition of the patient*’**. Responses: Yes / No / Insufficient evidence**
- **Preventability: If in your opinion the error resulted in an adverse event, to what extent was the adverse event preventable? Please use the grading below.**
    1. Virtually no evidence for preventability.
    2. Slight to modest evidence of preventability.
    3. Possibly preventable, but not very likely (less than 50–50, but close call).
    4. Probably preventable (more than 50–50, but close call).
    5. Strong evidence for preventability
    6. Virtually certain evidence of preventability.

For peer review only

**Describe each error in your own words. Please describe what should have happened AND what did happen.**	**Where did the error occur? (*Appendix 1.1*)**	**When did the error occur? (*Date, time*)**	**Category of error (*Appendix 1.2*)**	**Was the error associated with an adverse event? (*Y / N / insufficient evidence*)**	**To what extent was the adverse event preventable (*Appendix 1.3, grade 1-6*)?**
Example: "First dose of IV antibiotics should have been given immediately but was not given until three hours after prescribed"	*B*	*Date and time*	*B*	*N*	*n/a*
Example: Inappropriate administration of thrombolysis for an assumed minor pulmonary embolism, when in fact the patient had sustained a pulmonary haemorrhage.	*C*	*Date & time*	*A & B*	*Y*	*4*
Example: no vital signs / NEWS records were documented in the first 12 hours following transfer from the AMU to the ward. The patient suffered a cardiac arrest from which she could not be resuscitated.	*D*	*Date & time*	*F*	*Y*	*5*
Example: a single dose of a NSAID was given to this patient with AKI; the prescription was cancelled by the consultant the following morning.	*D*	*Date & time*	*B*	*N*	*n/a*

Describe each error in your own words. Please describe what should have happened AND what did happen.	Where did the error occur? (*Appendix 1.1*)	When did the error occur? (*Date, time*)	Category of error (*Appendix 1.2*)	Was the error associated with an adverse event? (*Y / N / insufficient evidence*)	To what extent was the adverse event preventable (*Appendix 1.3, grade 1-6*)?

Global assessment of quality of care
To what extent did this patient

How confident are you in your judgement of global quality? (select one only)
☐ Completely confident
☐ Substantially confident
☐ Partially confident
☐ Slightly confident
☐ Not at all confident

receive best practice care? (select one only)
• Completely
• Substantially
• Partially
• Very little
• Not at all

**To what extent was the adverse event preventable?**
1. Virtually no evidence for preventability.
2. Slight to modest evidence of preventability
3. Possibly preventable, but not very likely (less than 50–50, but close call)
4. Probably preventable (more than 50–50, but close call)
5. Strong evidence for preventability
6. Virtually certain evidence of preventability
n/a = not applicable, no adverse event

For peer review only

SPIRIT

STANDARD PROTOCOL ITEMS: RECOMMENDATIONS FOR INTERVENTIONAL TRIALS

SPIRIT 2013 Checklist: Recommended items to address in a clinical trial protocol and related documents*

Section/item	Item No	Description	Addressed on page number
**Administrative information**			
Title	1	Descriptive title identifying the study design, population, interventions, and, if applicable, trial acronym	Page1 of 37
Trial registration	2a	Trial identifier and registry name. If not yet registered, name of intended registry	See ref below
	2b	All items from the World Health Organization Trial Registration Data Set	________
Protocol version	3	Date and version identifier	V1
Funding	4	Sources and types of financial, material, and other support	Page 2 of 37
Roles and responsibilities	5a	Names, affiliations, and roles of protocol contributors	Page 1 of 37
	5b	Name and contact information for the trial sponsor	________
	5c	Role of study sponsor and funders, if any, in study design; collection, management, analysis, and interpretation of data; writing of the report; and the decision to submit the report for publication, including whether they will have ultimate authority over any of these activities	None
	5d	Composition, roles, and responsibilities of the coordinating centre, steering committee, endpoint adjudication committee, data management team, and other individuals or groups overseeing the trial, if applicable (see Item 21a for data monitoring committee)	Page 10 of 37

2a: https://www.journalslibrary.nihr.ac.uk/programmes/hsdr/1212817/#/

For peer review only - http://bmjopen.bmj.com/site/about/guidelines.xhtml

**Introduction**			
Background and rationale	6a	Description of research question and justification for undertaking the trial, including summary of relevant studies (published and unpublished) examining benefits and harms for each intervention	Page 4
	6b	Explanation for choice of comparators	Pages 4 & 5
Objectives	7	Specific objectives or hypotheses	Page 5
Trial design	8	Description of trial design including type of trial (eg, parallel group, crossover, factorial, single group), allocation ratio, and framework (eg, superiority, equivalence, noninferiority, exploratory)	Page 5
**Methods: Participants, interventions, and outcomes**			
Study setting	9	Description of study settings (eg, community clinic, academic hospital) and list of countries where data will be collected. Reference to where list of study sites can be obtained	Page 5
Eligibility criteria	10	Inclusion and exclusion criteria for participants. If applicable, eligibility criteria for study centres and individuals who will perform the interventions (eg, surgeons, psychotherapists)	Page 5
Interventions	11a	Interventions for each group with sufficient detail to allow replication, including how and when they will be administered	Page 5
	11b	Criteria for discontinuing or modifying allocated interventions for a given trial participant (eg, drug dose change in response to harms, participant request, or improving/worsening disease)	Not applicable
	11c	Strategies to improve adherence to intervention protocols, and any procedures for monitoring adherence (eg, drug tablet return, laboratory tests)	Not applicable
	11d	Relevant concomitant care and interventions that are permitted or prohibited during the trial	Not applicable
Outcomes	12	Primary, secondary, and other outcomes, including the specific measurement variable (eg, systolic blood pressure), analysis metric (eg, change from baseline, final value, time to event), method of aggregation (eg, median, proportion), and time point for each outcome. Explanation of the clinical relevance of chosen efficacy and harm outcomes is strongly recommended	Pages 7 & 8
Participant timeline	13	Time schedule of enrolment, interventions (including any run-ins and washouts), assessments, and visits for participants. A schematic diagram is highly recommended (see Figure)	Fig 1&2

For peer review only - http://bmjopen.bmj.com/site/about/guidelines.xhtml

Sample size	14	Estimated number of participants needed to achieve study objectives and how it was determined, including clinical and statistical assumptions supporting any sample size calculations	Page 9
Recruitment	15	Strategies for achieving adequate participant enrolment to reach target sample size	n/a
**Methods: Assignment of interventions (for controlled trials)**			
Allocation:			
Sequence generation	16a	Method of generating the allocation sequence (eg, computer-generated random numbers), and list of any factors for stratification. To reduce predictability of a random sequence, details of any planned restriction (eg, blocking) should be provided in a separate document that is unavailable to those who enrol participants or assign interventions	n/a
Allocation concealment mechanism	16b	Mechanism of implementing the allocation sequence (eg, central telephone; sequentially numbered, opaque, sealed envelopes), describing any steps to conceal the sequence until interventions are assigned	Page 7
Implementation	16c	Who will generate the allocation sequence, who will enrol participants, and who will assign participants to interventions	n/a
Blinding (masking)	17a	Who will be blinded after assignment to interventions (eg, trial participants, care providers, outcome assessors, data analysts), and how	Page 7
	17b	If blinded, circumstances under which unblinding is permissible, and procedure for revealing a participant's allocated intervention during the trial	n/a
**Methods: Data collection, management, and analysis**			
Data collection methods	18a	Plans for assessment and collection of outcome, baseline, and other trial data, including any related processes to promote data quality (eg, duplicate measurements, training of assessors) and a description of study instruments (eg, questionnaires, laboratory tests) along with their reliability and validity, if known. Reference to where data collection forms can be found, if not in the protocol	Pages 6&7
	18b	Plans to promote participant retention and complete follow-up, including list of any outcome data to be collected for participants who discontinue or deviate from intervention protocols	n/a

For peer review only - http://bmjopen.bmj.com/site/about/guidelines.xhtml

Data management	19	Plans for data entry, coding, security, and storage, including any related processes to promote data quality (eg, double data entry; range checks for data values). Reference to where details of data management procedures can be found, if not in the protocol	Fig 1
Statistical methods	20a	Statistical methods for analysing primary and secondary outcomes. Reference to where other details of the statistical analysis plan can be found, if not in the protocol	Page 8
	20b	Methods for any additional analyses (eg, subgroup and adjusted analyses)	Page 8 & 9
	20c	Definition of analysis population relating to protocol non-adherence (eg, as randomised analysis), and any statistical methods to handle missing data (eg, multiple imputation)	Page 8
**Methods: Monitoring**			
Data monitoring	21a	Composition of data monitoring committee (DMC); summary of its role and reporting structure; statement of whether it is independent from the sponsor and competing interests; and reference to where further details about its charter can be found, if not in the protocol. Alternatively, an explanation of why a DMC is not needed	www.hislac.org
	21b	Description of any interim analyses and stopping guidelines, including who will have access to these interim results and make the final decision to terminate the trial	n/a
Harms	22	Plans for collecting, assessing, reporting, and managing solicited and spontaneously reported adverse events and other unintended effects of trial interventions or trial conduct	Page 8
Auditing	23	Frequency and procedures for auditing trial conduct, if any, and whether the process will be independent from investigators and the sponsor	Page 7
**Ethics and dissemination**			
Research ethics approval	24	Plans for seeking research ethics committee/institutional review board (REC/IRB) approval	Ethics ref 13/WA/0372
Protocol amendments	25	Plans for communicating important protocol modifications (eg, changes to eligibility criteria, outcomes, analyses) to relevant parties (eg, investigators, REC/IRBs, trial participants, trial registries, journals, regulators)	Oversight committee

For peer review only - http://bmjopen.bmj.com/site/about/guidelines.xhtml

Consent or assent	26a	Who will obtain informed consent or assent from potential trial participants or authorised surrogates, and how (see Item 32)	n/a
	26b	Additional consent provisions for collection and use of participant data and biological specimens in ancillary studies, if applicable	n/a
Confidentiality	27	How personal information about potential and enrolled participants will be collected, shared, and maintained in order to protect confidentiality before, during, and after the trial	Anonymised
Declaration of interests	28	Financial and other competing interests for principal investigators for the overall trial and each study site	None
Access to data	29	Statement of who will have access to the final trial dataset, and disclosure of contractual agreements that limit such access for investigators	n/a
Ancillary and post-trial care	30	Provisions, if any, for ancillary and post-trial care, and for compensation to those who suffer harm from trial participation	n/a
Dissemination policy	31a	Plans for investigators and sponsor to communicate trial results to participants, healthcare professionals, the public, and other relevant groups (eg, via publication, reporting in results databases, or other data sharing arrangements), including any publication restrictions	Page 11
	31b	Authorship eligibility guidelines and any intended use of professional writers	n/a
	31c	Plans, if any, for granting public access to the full protocol, participant-level dataset, and statistical code	www.hislac.org
**Appendices**			
Informed consent materials	32	Model consent form and other related documentation given to participants and authorised surrogates	n/a
Biological specimens	33	Plans for collection, laboratory evaluation, and storage of biological specimens for genetic or molecular analysis in the current trial and for future use in ancillary studies, if applicable	n/a

*It is strongly recommended that this checklist be read in conjunction with the SPIRIT 2013 Explanation & Elaboration for important clarification on the items. Amendments to the protocol should be tracked and dated. The SPIRIT checklist is copyrighted by the SPIRIT Group under the Creative Commons "Attribution-NonCommercial-NoDerivs 3.0 Unported" license.

For peer review only - http://bmjopen.bmj.com/site/about/guidelines.xhtml

For peer review only
